# Fully Room Temperature Reprogrammable, Recyclable, and Photomobile Soft Actuators from Physically Cross-Linked Main-Chain Azobenzene Liquid Crystalline Polymers

**DOI:** 10.3390/molecules28104174

**Published:** 2023-05-18

**Authors:** Shengkui Ma, Lei Wang, Yan Zhou, Huiqi Zhang

**Affiliations:** State Key Laboratory of Medicinal Chemical Biology, Key Laboratory of Functional Polymer Materials (Ministry of Education), Collaborative Innovation Center of Chemical Science and Engineering (Tianjin), and College of Chemistry, Nankai University, Tianjin 300071, China; 1120170308@mail.nankai.edu.cn (S.M.); 1120190403@mail.nankai.edu.cn (L.W.); yzhou@jnxy.edu.cn (Y.Z.)

**Keywords:** azobenzene, main-chain liquid crystalline polymers, physically cross-linked, reprogrammable, recyclable, photomobile, room temperature

## Abstract

Fully room temperature three-dimensional (3D) shape-reprogrammable, recyclable, and photomobile azobenzene (azo) polymer actuators hold much promise in many photoactuating applications, but their development is challenging. Herein, we report on the efficient synthesis of a series of main-chain azo liquid crystalline polymers (LCPs) with such performances via Michael addition polymerization. They have both ester groups and two kinds of hydrogen bond-forming groups (i.e., amide and secondary amino groups) and different flexible spacer length in the backbones. Such poly(ester-amide-secondary amine)s (PEAsAs) show low glass transition temperatures (*T*_g_ ≤ 18.4 °C), highly ordered smectic liquid crystalline phases, and reversible photoresponsivity. Their uniaxially oriented fibers fabricated via the melt spinning method exhibit good mechanical strength and photoinduced reversible bending/unbending and large stress at room temperature, which are largely influenced by the flexible spacer length of the polymers. Importantly, all these fibers can be easily reprogrammed under strain at 25 °C into stable fiber springs capable of showing a totally different photomobile mode (i.e., unwinding/winding), mainly owing to the presence of low *T*_g_ and both dynamic hydrogen bonding and stable crystalline domains (induced by the uniaxial drawing during the fiber formation). They can also be recycled from a solution at 25 °C. This work not only presents the first azo LCPs with 3D shape reprogrammability, recyclability, and photomobility at room temperature, but also provides some important knowledge of their structure–property relationship, which is useful for designing more advanced photodeformable azo polymers.

## 1. Introduction

Photodeformable azobenzene (azo) polymers are smart materials capable of showing mobility under light irradiation [1,2,3,4,5,6,7,8,9,10,11,12,13]. They typically have cross-linked networks with well-aligned azo mesogens embedded inside. When they are exposed to an appropriate light, photoinduced stress is generated in the cross-linked polymer networks (normally on their thin surface layers with a thickness of about 1 μm due to the high light absorption of the *trans*-azo units [14,15]) owing to the photoisomerization of their azo units between the rod-like *trans*-form and bent *cis*-form. Such photomechanical stress can be accumulated and transported to the whole cross-linked networks, thus leading to their various macroscopic deformations such as bending/unbending, twisting/untwisting, and oscillation. Since the transformation of light energy into mechanical power is realized rapidly, precisely, and remotely, they are of great potential in a wide range of photodriven applications such as artificial muscles, soft robots, and microfluidic devices [1,2,3,4,5,6,7,8,9,10,11,12,13].

So far, a large number of photodeformable azo polymers have been developed via different strategies [1,2,3,4,5,6,7,8,9,10,11,12,13]. Among them, physically cross-linked photodeformable main-chain azo polymers have drawn rapidly increasing interest because of their apparent advantages over the chemically cross-linked ones, including not requiring complex chemical cross-linking procedures and high recyclability/reprocessability [11,16,17,18,19,20,21,22,23,24]. In addition, they also possess some obvious merits in comparison with side-chain systems, such as higher mechanical robustness and enhanced photomechanical effects, due to their more rigid backbone structures and much stronger chain anisotropy [1,5,25]. Over the past years, many main-chain azo polymers capable of forming physical cross-linking have been synthesized [11,16,17,18,19,20,21,22,23,24,26]. Our group has developed a series of physically cross-linked photodeformable main-chain azo liquid crystalline polymers (LCPs) or semi-crystalline polymers with supramolecular hydrogen-bonded networks via the versatile Michael addition polymerization [27,28], including those with both ester and secondary amino groups (i.e., poly(ester-secondary amine)s or PesAs) [16,18], those with both ester and amide groups (i.e., poly(ester-amide)s or PEAs) [17,20,21], and those with both ester and urea groups (i.e., poly(ester-urea)s or PEUs) [19] in their backbones. Their uniaxially oriented fibers fabricated via the melt spinning method exhibited photoinduced reversible bending/unbending even at (or close to) ambient temperatures. Particularly, we have recently prepared a series of high-molecular-weight main-chain azo semi-crystalline PEAs via Michael addition polymerization, which were readily processed into both physically cross-linked uniaxially oriented fibers and films with high mechanical robustness and photoinduced reversible bending/unbending at room temperature [20,21]. More importantly, they could be easily reprogrammed under strain at room temperature into various stable three-dimensional (3D) shapes (e.g., film helicoid/spiral ribbon and fiber spring) capable of showing highly reversible and totally different shape-dependent photomobile modes, which is attributed to their presence of low glass transition temperatures (*T*_g_ < room temperature) and both dynamic amide unit-induced hydrogen bonding and stable crystalline domains. Additionally, they also possessed high recyclability and reprocessability at room temperature. In this context, it is noteworthy that although some azo polymer actuators with 3D shape-reprogrammability and recyclability have been developed by introducing dynamic covalent bonds (DCBs) or hydrogen bonding interactions into their (cross-linked) structures, relatively high temperatures (≥90 °C) are normally needed to induce rearrangement/dissociation of DCBs [29,30,31] or the motion of polymer segments [22] for such a purpose, which may lead to their oxidation and degradation after repeated heating. It can thus be concluded that despite their great potential for facile and efficient large-scale fabrication of various photoactuators under mild ambient conditions, the development of fully room temperature 3D shape-reprogrammable, recyclable, and photomobile main-chain azo polymers remains challenging. Following our above work, it is important to check whether the physically cross-linked main-chain azo semi-crystalline polymers can be extended to other types of polymers (e.g., LCPs) for such a purpose. Moreover, more structure–property relationship knowledge still needs to be explored for rationally designing such advanced photomobile main-chain azo polymers.

**Scheme 1:**
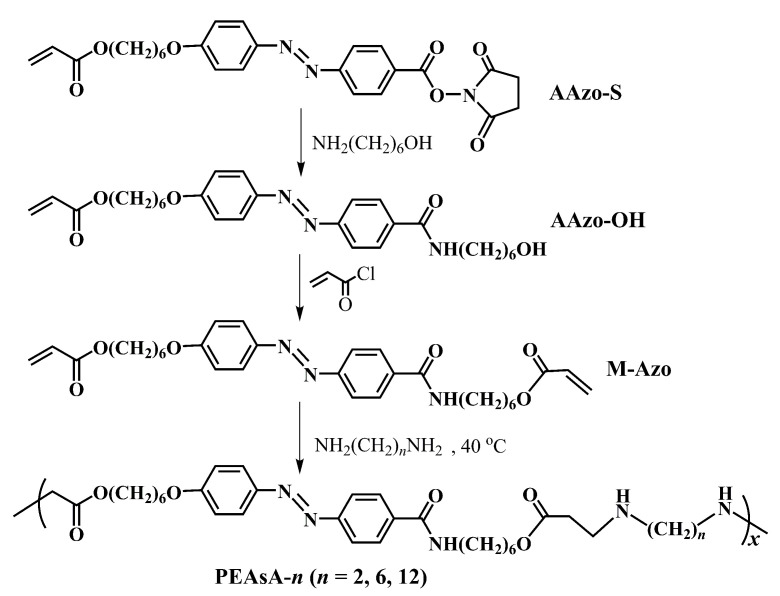
Synthetic routes and chemical structures of the diacrylate-type azo monomer bearing an amide group (M-Azo) and the main-chain azo liquid crystalline PEAsAs (prepared via the Michael addition polymerization of M-Azo and α,ω-alkanediamines (i.e., NH_2_(CH_2_)*_n_*NH_2_ (*n* = 2, 6, 12)).

Herein, we report on the efficient synthesis of a series of main-chain azo LCPs with both ester groups and two kinds of hydrogen bond-forming groups (i.e., both amide and secondary amino groups) in their backbones via Michael addition polymerization (Scheme 1) and their use for fabricating soft actuators with 3D shape reprogrammability, recyclability, and photomobility at room temperature. These poly(ester-amide-secondary amine)s (PEAsAs) show low *T*_g_ (<room temperature), a highly ordered smectic liquid crystalline phase, and reversible photoresponsivity. They can be readily processed via the melt spinning method into physically cross-linked uniaxially oriented fibers with good mechanical strength and photoinduced obvious and reversible bending/unbending and large stress at room temperature. The large influence of the flexible spacer length on the mechanical and photomechanical properties of these fibers was disclosed. In particular, both the easy 3D shape reprogrammability of the fibers under strain at room temperature into stable fiber springs (capable of showing a totally different photomobile mode (i.e., unwinding/winding)) and their high recyclability from a solution at room temperature were demonstrated. *To our knowledge, we report here the first successful example of azo LCPs that can be used to fabricate fully room temperature 3D shape-reprogrammable, recyclable, and photomobile soft actuators.*

## 2. Results and Discussion

### 2.1. Synthesis and Characterization of the Main-Chain Azo Liquid Crystalline Poly(Ester-Amide-Secondary Amine)s (PEAsAs)

The aim of this work includes the following two points: (i) to check whether physically cross-linked main-chain azo LCPs can be used for fabricating fully room temperature 3D shape-reprogrammable, recyclable, and photomobile soft actuators and (ii) to obtain knowledge of their structure–property relationship. To this end, we first prepared a diacrylate-type azo monomer with an amide group (i.e., M-Azo, Scheme 1) and then performed the Michael addition polymerization of M-Azo and three *α,ω*- alkanediamines (including 1,2-ethanediamine, 1,6-hexanediamine, and 1,12-dodecanediamine (i.e., NH_2_(CH_2_)*_n_*NH_2_, *n* = 2, 6, 12)) in DMF/methanol (3:1 *v*/*v*) at 40 °C. Homogeneous polymerization systems were achieved when the diamines were 1,2-ethanediamine and 1,6-hexanediamine, while the polymerization system became heterogeneous quickly (around 1 h) when the diamine was 1,12-dodecanediamine. Orange-yellow PEAsAs with different flexible spacers (i.e., PEAsA-*n* (*n* = 2, 6, 12)) were readily obtained in high yields (90–93%) at a polymerization time of 24 h (Table 1).

**Figure 1:**
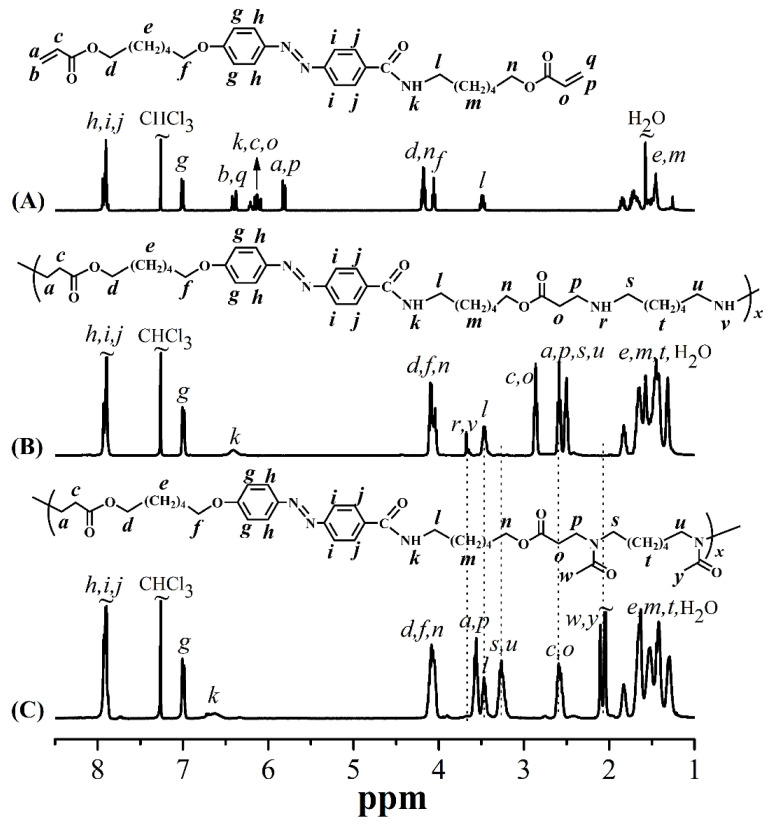
^1^H NMR spectra of M-Azo (**A**), PEAsA-6 (**B**), and A-PEAsA-6 (**C**) in CDCl_3_.

The azo monomer M-Azo, a representative azo polymer PEAsA-6, and the acetamide of PEAsA-6 (i.e., A-PEAsA-6, Scheme S2) were first characterized with ^1^H NMR (Figure 1A–C). It can be seen in Figure 1A,B that the proton signals from the acrylate groups of M-Azo are not present in the spectrum of PEAsA-6, confirming the absence of the azo monomer in the obtained polymer. The presence of the proton signals from secondary amino groups in Figure 1B (i.e., peaks *r,v*) reveals that PEAsA-6 is a main-chain linear polymer instead of a hyperbranched one with tertiary amino groups. This has been further verified by the fact that these secondary amino proton signals disappear in the spectrum of the acetamide of PEAsA-6, the proton signals from the methylene groups connected with the nitrogen atoms in PEAsA-6 (i.e., peaks *a*,*p* and *s*,*u* in Figure 1B,C) and those from the methylene groups connected with the carbonyl groups in PEAsA-6 and A-PEAsA-6 (i.e., peaks *c* and *o* in Figure 1B,C) show a large shift, and new proton signals can be found for the acetyl groups in A-PEAsA-6 (i.e., peaks *w* and *y* in Figure 1C). In addition, the chemical shifts and peak integrations of all the protons in PEAsA-6 agree with its expected structure. Both PEAsA-2 and PEAsA-12 also have similar ^1^H NMR characterization results. In addition to ^1^H NMR characterization, the ^13^C NMR spectra of M-Azo and PEAsA-6 were also measured (Figure S2B,C), which again confirms the successful synthesis of the desired azo monomer and polymer (as shown in Scheme 1).

GPC was used to characterize the molecular weights and molar-mass dispersities (*Đ*) of PEAsA-*n* (*n* = 2, 6, 12). Prior to GPC measurements, PEAsA-*n* were reacted with acetic anhydride to eliminate the strong interactions between their secondary amino groups and GPC columns [16]. The number-average molecular weights of the acetamides of PEAsA-*n* (i.e., A-PEAsA-*n* (*n* = 2, 6, 12)) determined by GPC (*M*_n,GPC_) range from 8260 to 13,300 g/mol, and their *Đ* values are between 1.23 and 1.98 (Table 1).

**Figure 2:**
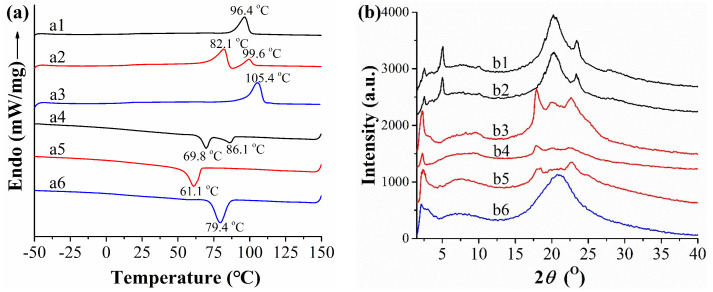
(**a**) DSC thermograms of PEAsA-*n* (*n* = 2 (a1,a4), 6 (a2,a5), 12 (a3,a6)) from the second heating scan (a1–a3) and first cooling scan (a4–a6) (±10 °C min^−1^). (**b**) XRD spectra of PEAsA-*n* (*n* = 2, 6, 12) (first cooling/second heating rate: ±10 °C min^−1^): (b1,b2) XRD spectra of PEAsA-2 obtained after being annealed at 84 °C (b1) and 68 °C (b2) for 30 min (during the first cooling process); (b3–b5) XRD spectrum of PEAsA-6 obtained after being annealed at 59 °C (b3) (during the first cooling process), as well as those obtained after being annealed at 80 °C (b4) and 95 °C (b5) for 30 min (during the second heating process); (b6) XRD spectrum of PEAsA-12 obtained after being annealed at 77 °C for 30 min (during the first cooling process).

The thermal stability and phase transition behaviors of PEAsA-*n* (*n* = 2, 6, 12) were then characterized with TGA, DSC, POM, and XRD. TGA analyses revealed that PEAsA-*n* have high thermal stability (with temperatures of 5% weight losses of 260, 275, and 289 °C for *n* = 2, 6, and 12, respectively) (Table 1). The DSC thermograms of PEAsA-*n* show one rather weak glass transition step (only during their second heating scans) and one or two phase transition peak(s) (during their first cooling and second heating scans) (Figure 2a). Their *T*_g_ values are 18.4, 17.3, and 16.8 °C for *n* = 2, 6, and 12, respectively, which are all lower than the ambient temperature (25 °C). POM characterization results confirmed the presence of enantiotropic (broken) focal-conic fan-shaped liquid crystalline textures during the phase transition processes of PEAsA-*n* (Figure S3). The XRD spectra of PEAsA-*n* exhibited some sharp diffraction peaks in both small-angle areas (2*θ* < 5°) and wide-angle areas (2*θ* around 22.5°) (Figure 2b), suggesting the presence of a highly ordered smectic liquid crystalline phase [32,33,34]. The above results demonstrate that PEAsA-*n* (*n* = 2, 6, 12) are LCPs of highly ordered smectic structures. Therefore, the phase transition peaks in the DSC thermograms of PEAsA-12 (Figure 2(a3,a6)), those in the DSC second heating scan thermogram of PEAsA-2 (Figure 2(a1)), and those in the DSC first cooling scan thermogram of PEAsA-6 (Figure 2(a5)), represent the transition between the highly ordered smectic liquid crystalline and isotropic phases. The presence of two phase transition peaks in both the DSC first cooling scan thermogram of PEAsA-2 (Figure 2(a4)) and the DSC second heating scan thermogram of PEAsA-6 (Figure 2(a2)) might be ascribed to their presence of different ordered (highly ordered smectic) liquid crystalline structures because no discernible difference was observed between their XRD spectra before or after the first peak and those between the two peaks (Figure 2(b1,b2,b4,b5)).

**Figure 3:**
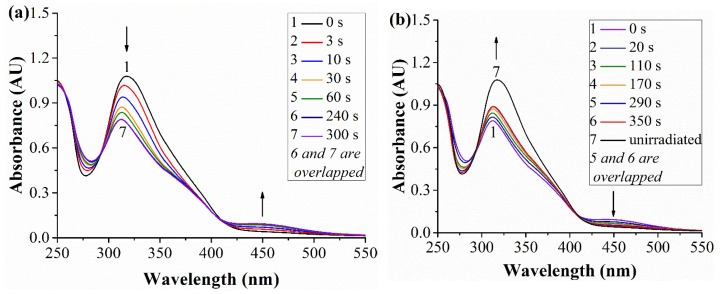
UV-vis spectral changes in dependence of time for the thin PEAsA-6 film (cast on the quartz glass plate, film thickness: 740 nm) upon its exposure to 365 nm of UV light (90 mW cm^−2^) (**a**) and upon irradiating the polymer film at the photostationary state with visible light (λ > 510 nm, 35 mW cm^−2^) (**b**) at 25 °C.

A UV-vis spectrometer was used to study the photoresponsivity of a thin PEAsA-6 film (Figure 3). The UV-vis spectra of the thin PEAsA-6 film show one strong π-π* transition band around 315 nm (from the *trans*-azo isomer) and one rather weak n-π* transition band around 450 nm (from the *cis*-azo isomer) [35]. The thin PEAsA-6 film exhibited a simultaneous decrease in the absorption band around 315 nm and an increase in the absorption band around 450 nm upon exposure to 365 nm of UV light until its photostationary state was achieved after 240 s, indicating the occurrence of the *trans* to *cis* photoisomerization of its azo units (Figure 3a). Exposure of the above PEAsA-6 film in the photostationary state to visible light (*λ* > 510 nm) led to the *cis* to *trans* photoisomerization of the azo units (Figure 3b). However, only part of the *trans*-isomer (83%) was recovered when the new photostationary state was reached, which was previously reported by us [16,36,37] and others [38,39,40]. Nevertheless, thin PEAsA-6 film showed highly reversible *trans*/*cis* photoisomerization under the alternative UV and visible light irradiation (Figure S4).

### 2.2. Mechanical and Photomechanical Properties of the Uniaxially Oriented PEAsA-n Fibers

**Figure 4:**
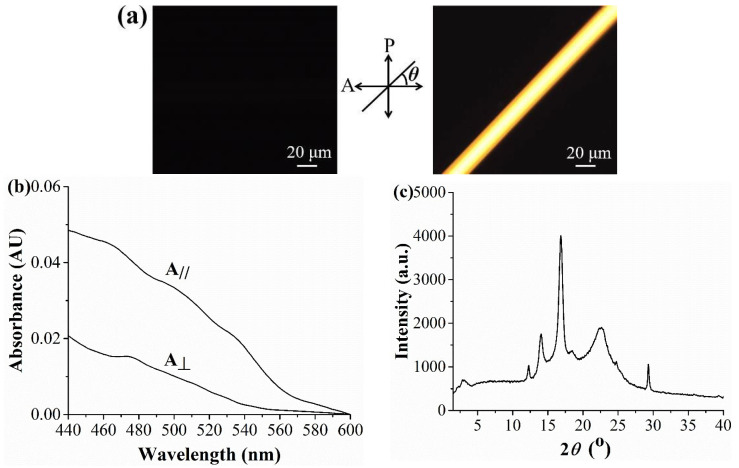
(**a**) POM images of the textures of a PEAsA-6 fiber taken at room temperature. Sample angle to the analyzer: *θ* = 0° (left) and 45° (right). (**b**) Polarized UV-vis absorption spectra of the uniaxially oriented PEAsA-6 fiber. (**c**) XRD spectrum of the uniaxially oriented PEAsA-6 fiber.

In this section, the uniaxially oriented fibers of PEAsA-*n* (*n* = 2, 6, 12) were fabricated, and their mechanical and photomechanical properties were studied. We first prepared the fibers of PEAsA-*n* (*n* = 2, 6, 12) via the simple melt spinning method following our previously reported approach [16,17,18,19,20,21]. They showed homogeneous azo mesogen alignment along the fiber axes, as revealed by a sharp contrast inversion every 45° upon rotating the samples with respect to the analyzer under the crossed POM observation (Figure 4a and Figure S5) [16,17,18,19,20,21,41,42]. The order parameters of PEAsA-*n* fibers were determined to be 0.46, 0.43, and 0.39 (for *n* = 2, 6, and 12, respectively) by measuring their polarized UV-vis absorption spectra (Figure 4b and Figure S6) [43,44,45]. The XRD characterization results indicated the presence of crystalline structures in PEAsA-*n* fibers (Figure 4c and Figure S7), which should be induced by the uniaxial drawing during the fiber-forming processes [46,47]. These uniaxially oriented azo polymer fibers should also have physically cross-linked supramolecular hydrogen-bonded networks due to the presence of amide and secondary amino groups in the polymer backbones [16,17].

**Figure 5:**
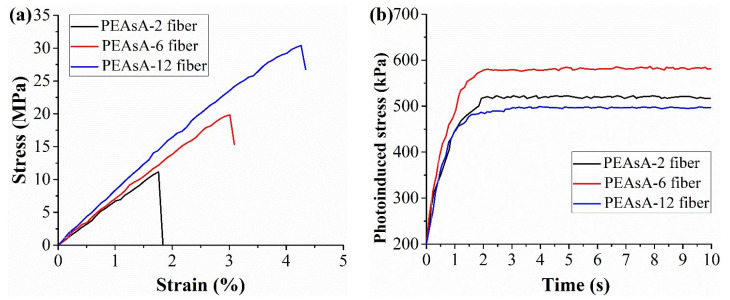
(**a**) Stress–strain curves of the uniaxially oriented PEAsA-*n* fibers (length: 10 mm, diameter: 20 μm) measured at 25 °C at a stretching rate of 10 mm min^−1^. (**b**) Dependence of the photoinduced stress of the uniaxially oriented PEAsA-*n* fibers (length: 10 mm, diameter: 25–30 μm) on the irradiation time of 365 nm of UV light (90 mW cm^−2^) at 25 °C. An external stress of 200 kPa was initially loaded on the fibers to keep their length constant.

Figure 5a presents the stress–strain curves of the uniaxially oriented PEAsA-*n* (*n* = 2, 6, 12) fibers, which were determined by using an automatic single fiber tensile strength tester at an ambient temperature. PEAsA-*n* fibers show an elastic modulus (*G*) of 656.2 ± 26.1, 704.4 ± 35.3, and 749.3 ± 11.4 MPa, a rupture strength (*δ*_b_) of 10.9 ± 0.6, 20.9 ± 0.9, and 30.7 ± 0.4 MPa, and a breaking strain of 1.8 ± 0.1%, 3.0 ± 0.1%, and 4.3 ± 0.1% for *n* = 2, 6, and 12, respectively (Table 2). It is somewhat surprising to see that all the mechanical parameters of the above fibers increase with an increase in their flexible spacer length, although their hydrogen bonding densities and order parameters follow the reverse trend. The real reason for this phenomenon is not very clear but might be related to the different aggregation states of these fibers [20,21]. Further investigation is ongoing to provide a reasonable explanation.

**Figure 6:**
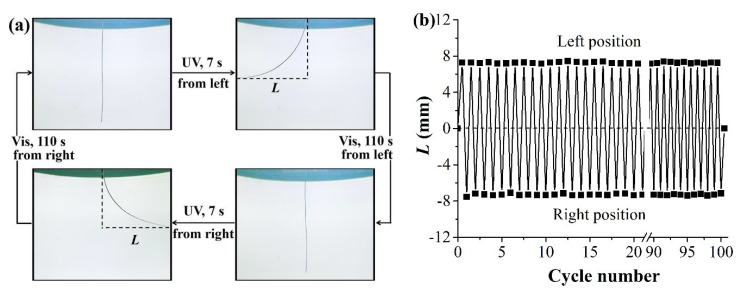
(**a**) Photographs of a representative PEAsA-6 fiber that exhibits photoinduced bending and unbending upon irradiation with 365 nm of UV light (90 mW cm^−2^) and visible light (λ > 510 nm, 35 mW cm^−2^) at 25 °C (fiber size: 10 mm × 20 μm). (**b**) The reversible deformation of the PEAsA-6 fiber characterized by tracing the bent distance from its straight state at 25 °C.

The photodeformation behaviors of the uniaxially oriented PEAsA-*n* fibers were then studied by first pasting part of a representative PEAsA-6 fiber onto an aluminum block, heating it to 25 °C by a hot stage, and then exposing it to 365 nm of UV (90 mW cm^−2^) and visible light (*λ* > 510 nm, 35 mW cm^−2^). The fiber bent to the left side toward the incident light source along the fiber axis when it was exposed to 365 nm of UV light from the left side of the fiber, and its maximum bending was reached within 7 s (Figure 6a). The bent fiber could restore its initial straight state within 110 s under the irradiation of visible light from the left side. The PEAsA-6 fiber showed excellent fatigue resistance, as demonstrated by its highly repeatable photomobile behavior (over 100 cycles) and almost constant bending/unbending times and bending angles (Figure 6b). Moreover, the bending direction of the fiber could also be directly controlled by changing the irradiation direction of the light source, thus allowing the well-controlled 3D photomobility of the fiber on demand. Similar to the PEAsA-6 fiber, the uniaxially oriented PEAsA-2 and PEAsA-12 fibers also showed obvious and reversible photoinduced bending and unbending behaviors under the same photoirradiation conditions (Figure S8).

Next, the UV light-induced mechanical stress in the uniaxially oriented PEAsA-*n* fibers was determined by using a mechanical testing instrument in the stretching mode under an external tension at 25 °C. Three parallel fibers were fixed by clamping their two ends, and an initial stress of 200 kPa was loaded onto them to keep their length constant. Figure 5b shows an obvious rapid increase in the stress for all fibers upon their exposure to 365 nm of UV light (90 mW cm^−2^), indicating the generation of photoinduced stress in the fibers. The maximum stress generated in PEAsA-*n* fibers was quickly reached within about 2 s in all cases, which was 320.4 ± 17.5, 373.2 ± 22.0, and 295.6 ± 16.3 kPa for PEAsA-*n* fibers with *n* = 2, 6, and 12, respectively (Table 2). A much higher photoinduced stress was observed for the PEAsA-6 fiber in comparison with PEAsA-2 and PEAsA-12 fibers. This phenomenon might be ascribed to the presence of the optimal supramolecular hydrogen-bonded physical cross-linking networks in the PEAsA-6 fiber (while PEAsA-2 and PEAsA-12 fibers might have cross-linking networks that are too tight and too loose, respectively), which might impart the PEAsA-6 fiber with the best synergistic effect of the mobility of both the azo units and polymer networks and thus the higher photoinduced stress. These fibers show much larger photoinduced stress than our previously reported physically cross-linked main-chain azo liquid crystalline poly(ester-secondary amine)s (one of this kind of polymer fiber generated a maximum stress of 240 kPa under the much stronger 365 nm of UV light irradiation (150 mW cm^−2^) [16]). In particular, their photoinduced stress is close to the stress generated by the chemically cross-linked azo LCP films [48] and fibers [42], as well as that of the human muscles (around 300 kPa) [42,48].

**Figure 7:**
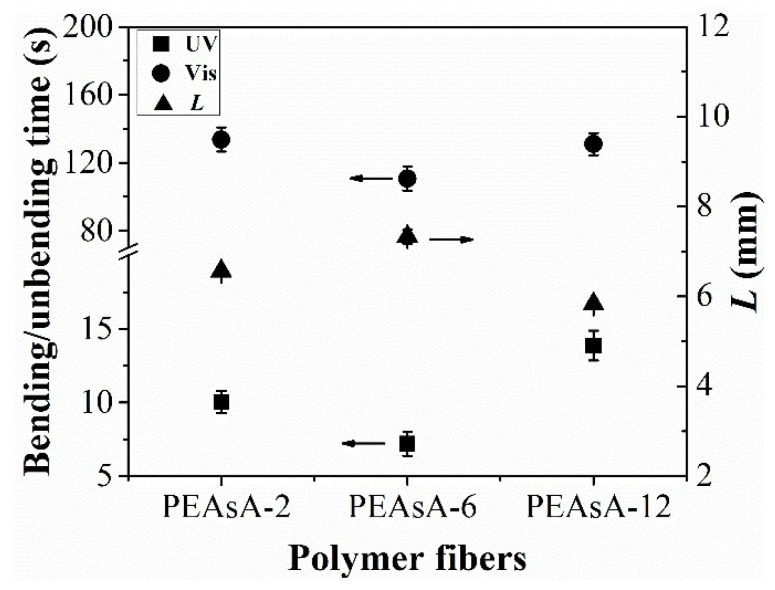
Dependence of the UV light-induced bending times and visible light-induced unbending times (for reaching the maximum bending and restoring the initial straight state, respectively) and bending amplitudes (*L*) of PEAsA-*n* fibers on the flexible spacer length in the polymer backbones.

It has been well-established that the photoinduced bending/unbending rates and bending amplitudes of the uniaxially oriented azo polymer fibers are determined by the combined effect of their rigidity (or elastic moduli) and photoinduced stress [21]. The photoinduced bending/unbending rates and bending amplitudes of the azo polymer fibers typically increase with a decrease in their rigidity or an increase in their photoinduced stress. It can be seen in Figure 7 that the PEAsA-6 fiber shows the largest bending/unbending rates and bending amplitude among the studied fibers, which could be attributed to the combined effect of its moderate modulus and the largest photoinduced stress. Additionally, the PEAsA-2 fiber exhibits a somewhat larger bending rate and bending amplitude than the PEAsA-12 fiber because of its lower modulus and larger photoinduced stress than the PEAsA-12 fiber.

### 2.3. Room Temperature 3D Shape Reprogrammability and Recyclability of PEAsA-n Fibers

**Figure 8:**
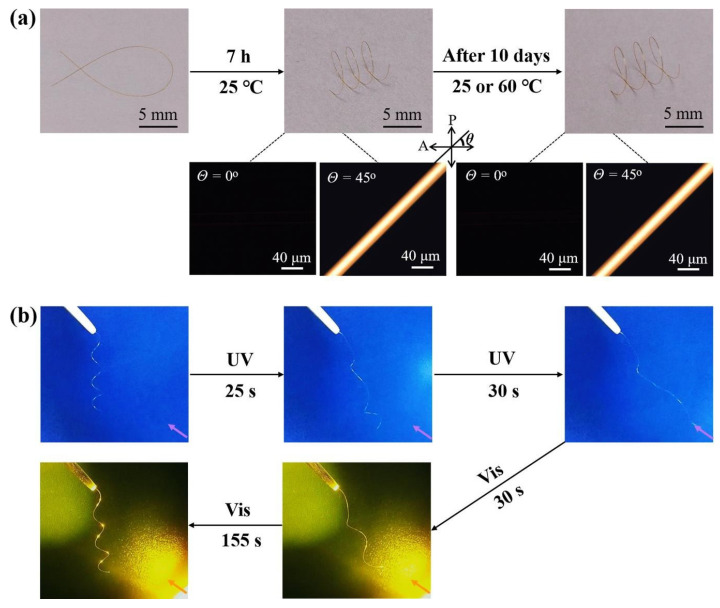
Three-dimensional shape reprogramming of a uniaxially oriented PEAsA-6 fiber and photomobile behaviors of the resulting reshaped photoactuator. (**a**) Reshaping a uniaxially oriented PEAsA-6 fiber (diameter: 24 μm) into a fiber spring (the wire diameter: 5 mm) at 25 °C and its shape (top) and homogeneous azo mesogen alignment (below, the reshaped fiber was untwisted for POM observation) remain unchanged after being kept at 25 or 60 °C for 10 days. (**b**) Photomobile behaviors of the reshaped fiber spring at room temperature under the irradiation of UV light (365 nm, 40 mW cm^−2^) and visible light (λ > 510 nm, 30 mW cm^−2^).

The 3D shape reprogrammability of the uniaxially oriented PEAsA-*n* (*n* = 2, 6, 12) fibers was studied. To our delight, stable 3D-shaped photoactuators could be easily obtained by reprograming all the fibers fabricated from the main-chain azo LCPs (i.e., PEAsA-*n*) under strain at room temperature, similar to as our previously reported main-chain azo semi-crystalline poly(ester-amide)s [20]. We started to reprogram the 3D shape of a linear PEAsA-6 fiber. A fiber spring with a wire diameter of 5 mm was readily obtained by reshaping the PEAsA-6 fiber under strain at 25 °C (Figure 8a). Upon exposure to 365 nm of UV light (40 mW cm^−2^), the above-obtained fiber spring exhibited obvious unwinding until an almost straight state was eventually reached after 55 s (Figure 8b and Movie S1). The fiber was then irradiated to visible light (λ > 510 nm, 30 mW cm^−2^), which resulted in the winding of the fiber to its original spring state after an irradiation time of 185 s. The UV light-induced unwinding and visible light-induced winding behaviors of this reshaped fiber proved to be highly reversible. They could be repeated over 100 cycles with the unwinding/winding times and amplitudes remaining almost constant, suggesting the excellent fatigue resistance of the fiber spring. Particularly, the reshaped fiber spring showed negligible change in both its shape and homogeneous azo alignment (Figure 8a) and its photomobile behaviors after it was kept at both 25 and 60 °C for 10 days. These results are quite similar to those of our previously reported reshaped main-chain azo semi-crystalline PEA fiber spring [20].

Similarly, the uniaxially oriented PEAsA-2 and PEAsA-12 fibers could also be reprogrammed under strain at room temperature into fiber springs with high stability and highly reversible unwinding/winding photomobile behaviors (Figures S9 and S10, Movies S2 and S3). In addition, similar to the linear fibers, the reshaped PEAsA-6 fiber spring also exhibits more rapid photoinduced unwinding/winding than the reshaped PEAsA-2 and PEAsA-12 fiber springs under the same photoirradiation conditions. The easy room temperature 3D shape reprogrammability of PEAsA-*n* fibers is attributable to the presence of dynamic physical cross-linking networks and low *T*_g_ (< room temperature), while the high stability of the reshaped actuators can be ascribed to the existence of stable crystalline domains [20], as revealed by XRD characterization results (Figure 4c and Figure S7).

Next, the high recyclability and reprocessability of our uniaxially oriented fibers were demonstrated. For example, PEAsA-6 fibers could be readily recycled by first dissolving the original fibers into chloroform, evaporating the solvent to dryness, and then drying under a vacuum (all the above procedures were performed at room temperature). This simple room temperature recycling capability of our PEAsA-based photoactuator represents a distinct advantage in comparison with photoactuators that have to be recycled by heating and reversible chemical reactions, mainly because the polymer can be kept intact under such a mild recycling process (Figure S11). In addition, the recycled PEAsA-6 can be further reprocessed via the melt spinning method into the uniaxially oriented fibers capable of showing reversible photoinduced bending/unbending with high fatigue resistance (more than 100 cycles), similar to as the original fiber.

## 3. Materials and Methods

### 3.1. Materials and Reagents

Tetrahydrofuran (THF, Tianjin Chemical Reagent Co., Ltd., Tianjin, China, analytical grade (AR)) was refluxed over sodium and then distilled. *N,N*-Dimethylformamide (DMF, Tianjin Jiangtian Chemical Technology Co., Ltd., Tianjin, China, AR) was dried with anhydrous magnesium sulfate and then distilled under a vacuum. Triethylamine (TEA, Tianjin Jiangtian Chemical Technology Co., Ltd., AR) was dried with anhydrous sodium sulfate and then distilled. Methanol (Tianjin Jiangtian Chemical Technology Co., Ltd., AR) and chloroform (CHCl_3_, Tianjin Chemical Reagent Co., Ltd., AR) were refluxed over calcium hydride and then distilled. *N*-Hydroxysuccinimide 4-((4-(*ω*-acryloyloxyhexyloxy))phenylazo)benzoate (AAzo-S, Scheme 1) was synthesized according to our previously reported procedure (Scheme S1) [36]. 6-Amino-1-hexanol (97%), acryloyl chloride (98%), 1,2-ethanediamine (99%), 1,6-hexanediamine (96%), 1,12-dodecanediamine (98%), acetic anhydride (AR), diethyl ether (AR), dichloromethane (CH_2_Cl_2_, AR), and all the other chemicals were commercially available and used directly without further purification.

### 3.2. Synthesis of AAzo-OH (Scheme 1)

To a solution of AAzo-S (3.00 g, 6.08 mmol) in dried DMF (30 mL) was added dropwise a solution of 6-amino-1-hexanol (0.74 g, 6.08 mmol) in dried methanol (10 mL). The reaction mixture was magnetically stirred at 40 °C for 24 h in the dark, cooled down to room temperature, and then poured into cold distilled water. The precipitate was collected by filtration, washed thoroughly with distilled water, and then dried at 40 °C under a vacuum to provide the desired orange-yellow AAzo-OH (yield: 97%). UV-vis (DMF): *λ*_max_/nm (ε/L mol^−1^ cm^−1^) = 360 (17,540) and around 450 (not available due to the overlap of the absorption bands). ^1^H NMR (400 MHz, CDCl_3_): *δ* (ppm) = 8.02–7.83 (m, 6H, Ar-H), 7.08–6.95 (d, 2H, Ar-H), 6.46–6.35 (d, 1H, CH=C-COO-), 6.28–6.18 (t, 1H, -CONH-), 6.17–6.06 (dd, 1H, C=CH-COO-), 5.87–5.77 (d, 1H, CH=C-COO-), 4.26–4.14 (t, 2H, -CH_2_OAr-), 4.12–3.99 (t, 2H, -COOCH_2_-), 3.70–3.60 (q, 2H, -CH_2_-OH), 3.55–3.42 (q, 2H, -CONCH_2_-), and 1.97–1.20 (m, 16H, 2[-(CH_2_)_4_-]). ^13^C NMR (400 MHz, CDCl_3_): *δ* (ppm) = 167.17, 166.46, 162.19, 154.45, 146.91, 135.91, 130.71, 128.63, 127.96, 125.19, 122.66, 114.85, 68.26, 64.59, 62.66, 40.11, 32.60, 29.69, 29.13, 28.63, 26.69, 25.82, 25.80, and 25.42.

### 3.3. Synthesis of the Diacrylate-Type AZO Monomer with an Amide Group (M-Azo, Scheme 1)

A solution of acryloyl chloride (0.68 mL, 8.00 mmol) in dried THF (32 mL) was added dropwise under magnetic stirring into a cooled (0 °C) mixed solution of AAzo-OH (1.98 g, 4.00 mmol) and dried TEA (1.12 mL, 8.00 mmol) in a mixture of dried THF (44 mL) and DMF (8 mL). After the reaction mixture was magnetically stirred first at 0 °C for 1 h and then at room temperature for 24 h, it was filtered to remove TEA hydrochloride. The solvents of the filtrate were removed by using a rotary evaporator under a vacuum, and the resulting crude product was purified with silica gel column chromatography by using a mixture of CH_2_Cl_2_ and methanol (100:1, *v*/*v*) as the eluent to provide the orange M-Azo (yield: 78%). UV-vis (DMF): *λ*_max_/nm (ε/L mol^−1^cm^−1^) = 360 (20,620) and around 450 (not available due to the overlap of the absorption bands). ^1^H NMR (400 MHz, CDCl_3_): *δ* (ppm) = 8.01–7.86 (m, 6H, Ar-H), 7.06–6.97 (d, 2H, Ar-H), 6.46–6.35 (d, 2H, 2CH=C-COO-), 6.25–6.18 (t, 1H, -CONH), 6.17–6.07 (dd, 2H, 2C=CH-COO-), 5.86–5.78 (d, 2H, 2CH=C-COO-), 4.22–4.13 [m, 4H, 2(-COOCH_2_-)], 4.10–4.02 (t, 2H, -CH_2_Oar-), 3.53–3.44 (m, 2H, Ar- CONCH_2_-), and 1.90–1.20 (m, 16H, 2[-(CH_2_)_4_-]). ^13^C NMR (400 MHz, CDCl_3_): *δ* (ppm) = 166.99, 166.35, 162.10, 154.33, 146.83, 135.91, 130.63, 128.57, 127.93, 125.11, 122.56, 114.77, 68.18, 64.50 (64.44), 40.07, 29.55, 29.06, 28.55, 26.60, 25.74, and 25.64.

### 3.4. Synthesis of the Main-Chain Azo LCPs with Both Ester Groups and Two Kinds of Hydrogen Bond-Forming Groups (i.e., Both Amide and Secondary Amino Groups) and Different Lengths of Flexible Spacers in Their Backbones (PEAsA-n (n = 2, 6, 12), Scheme 1)

PEAsA-6 was prepared via the Michael addition polymerization of M-Azo and 1,6- hexanediamine in a mixture of DMF and methanol (3:1 *v*/*v*) as follows. To a solution of M-159 Azo (500.0 mg, 0.91 mmol) in DMF (15 mL) was added dropwise a solution of 1,6-hex-160 anediamine (110.0 mg, 0.91 mmol) in methanol (5 mL). The reaction mixture was magnetically stirred at 40 °C for 24 h, cooled to room temperature, and then poured into cold diethyl ether (200 mL). The precipitate was filtered, washed with diethyl ether thoroughly, and then dried at 40 °C under a vacuum to a constant weight, leading to the orange-yellow PEAsA-6 (yield: 92%) (Table 1).

PEAsA-2 and PEAsA-12 were synthesized similar to PEAsA-6 in a yield of 93% and 90%, respectively (Table 1).

### 3.5. Polymer Analogous Reactions of PEAsA-n with Acetic Anhydride (Scheme S2)

The polymer analogous reaction of PEAsA-6 with acetic anhydride is presented as follows. A solution of PEAsA-6 (20.0 mg, containing 0.03 mmol of repeat unit) and acetic anhydride (230.0 μL, 2.4 mmol) in dried CHCl_3_ (2 mL) was magnetically stirred at 25 °C for 5 h and then poured into diethyl ether (20 mL). The precipitate was collected through centrifugation, redissolved in CHCl_3_, and then precipitated into diethyl ether again. After this purification procedure was repeated once, the solid product was dried at 40 °C under a vacuum to a constant weight, leading to the orange acetamide of PEAsA-6 (i.e., A-PEAsA-6) (yield: 92%).

The acetamides of PEAsA-2 and PEAsA-12 (i.e., A-PEAsA-2 and A-PEAsA-12) were prepared similar to A-PEAsA-6 in a yield of 94% and 93%, respectively.

### 3.6. Fabrication of the Uniaxially Oriented Main-Chain Azo Polymer Fibers

The uniaxially oriented PEAsA-6 fibers were prepared via the melt spinning method following our previously reported method [16,17,18,19,20,21]. A total of 2 mg of PEAsA-6 was added onto a clean glass slide, which was heated to 110 °C on a hot stage (IKA, C-MAG HP7) to obtain a melted sample. The desired azo polymer fibers were then obtained by dipping the tip of a metallic tweezer into the melted sample and pulling it quickly.

The uniaxially oriented PEAsA-2 fibers were prepared similar to PEAsA-6 fibers. The uniaxially oriented PEAsA-12 fibers were prepared by adding 2 mg of PEAsA-12 onto a clean glass slide, heating it to 130 °C and keeping it at that temperature for 1 min, lowering the temperature to 125 °C, dipping the tip of a metallic tweezer into the melted sample, and then pulling it quickly.

### 3.7. 3D Shape Reprogramming of the Uniaxially Oriented PEAsA-n Fibers

A uniaxially oriented PEAsA-6 fiber (diameter: 24 μm) was reshaped by being rolled around a glass rod (diameter: 5 mm) and then kept at 25 °C for 7 h to obtain the fiber spring.

PEAsA-2 and PEAsA-12 fibers were reshaped similar to the PEAsA-6 fiber to obtain their corresponding fiber springs.

### 3.8. Recycling of PEAsA-6 Fibers

PEAsA-6 fibers were recycled by first dissolving them into CHCl_3_, evaporating the solvent to dryness, and then drying under a vacuum to a constant weight (all procedures were performed at room temperature). The recycled product was then characterized by using ^1^H NMR.

### 3.9. Characterization

The samples were characterized with ^1^H NMR (^13^C NMR), a thermogravimetric analyzer (TGA, Netzsch TG 209), a differential scanning calorimeter (DSC, Netzsch 200 F3), a polarized optical microscope (POM, Olympus BX51 POM (with a crossed polarizer and analyzer) equipped with a Linksys 32 THMSE600 hot stage and a digital camera (Micropublisher 5.0 RTV)), a UV-vis scanning spectrophotometer (TU1900, Beijing Purkinje General Instrument Co., Ltd., Beijing, China), and an X-ray diffractometer (XRD, Rigaku SmartLab 9KW) following our previously described procedures [20,21]. The detailed preparation procedure of the thin polymer film for the photoresponsivity study and the detailed sample preparation methods for the XRD characterization of both the azo polymer powders and the uniaxially oriented azo polymer fibers (Figure S1) are described in the Supporting Information.

The molecular weights and molar-mass dispersities (*Ð*) of the polymers were determined with a GPC, and the order parameters (*S*) of the uniaxially oriented PEAsA-*n* fibers were obtained by measuring their polarized absorption spectra by using a UV-vis scanning spectrophotometer equipped with a Glan–Taylor prism attachment. The instrument information and measurement details are presented in the Supporting Information.

The photomobile behaviors of the uniaxially oriented polymer fibers were studied at 25 °C as follows. The polymer fibers were pasted onto an aluminum block, whose temperature was adjusted to 25 °C by using a hot stage under the control of a thermocouple (IKA CMAG HP7). The polymer fibers were then exposed to 365 nm of UV light (90 mW cm^−2^) or visible light (*λ* > 510 nm, 35 mW cm^−2^) (both of them obtained using different glass filters) emitted from a high-pressure mercury lamp (USHIO SP-7). A CCD camera (KBier-1202) was used to record the photoresponsivity of the polymer fibers.

The photomobile behaviors of the reshaped fiber springs were studied by irradiating them with 365 nm of UV light (40 mW cm^−2^) and visible light (*λ* > 510 nm, 30 mW cm^−2^) from a Warsun Series R838 light source (a glass filter was used to obtain the desired visible light) at 25 °C. Their photomobile images and movies were recorded using a mobile phone camera.

An automatic single-fiber tensile strength tester (LLY-06E, Laizhou, China) was used to study the mechanical properties of the uniaxially oriented polymer fibers at room temperature. Polymer fibers were stretched at a speed of 10 mm min^−1^ during tensile testing. Three fibers of each sample were tested, and their average mechanical parameters were presented. The sizes of the PEAsA-*n* fibers are 10 mm × 20 μm (length × diameter).

An HY-0580 mechanical testing instrument (Shanghai Hengyi Precision Instruments Co., Ltd., Shanghai, China) was utilized to measure the photoinduced stress of the uniaxially oriented PEAsA-*n* fibers in the stretching mode under an external tension at 25 °C. Three parallel fibers were fixed by clamping their two ends. An initial stress of 200 kPa was loaded onto the fibers to keep their length constant. The time dependence of the photoinduced stress in the fibers upon exposure to 365 nm of UV light (90 mW cm^−2^) was recorded. Three measurements were repeated for each sample and their averages were used for analyses. The sizes of the azo polymer fibers are 10 mm × (25–30) μm (length × diameter).

## 4. Conclusions

We have demonstrated for the first time the efficient synthesis of a series of main-chain azo liquid crystalline poly(ester-amide-secondary amine)s (PEAsAs) with low *T*_g_ (<room temperature) and a highly ordered smectic phase via Michael addition polymerization and their use for fabricating fully room temperature reprogrammable, recyclable, and photomobile soft actuators. Their physically cross-linked uniaxially oriented fibers (fabricated via the melt spinning method) show good mechanical strength, as well as photoinduced obvious and reversible bending/unbending and large stress at room temperature, which are influenced by the flexible spacer length of the polymers. In particular, such uniaxially oriented fibers can be easily reprogrammed under strain at room temperature into stable 3D-shaped fiber springs with highly reversible photoinduced unwinding/winding behaviors. Additionally, the high room temperature recyclability of the azo polymer fibers was also demonstrated. It is believable that such physically cross-linked main-chain azo LCPs hold much promise in conveniently fabricating various advanced photoactuators, such as artificial muscles and smart textiles. We also believe that the structure–property relationship knowledge obtained in this work will promote the further rational development of more advanced fully room temperature reprogrammable, recyclable, and photomobile main-chain azo polymers.

## Figures and Tables

**Table 1:** Synthetic and characterization data of the main-chain azo liquid crystalline PEAsAs.

Sample	Yield (%)	*M*_n,GPC_ (g/mol) *^a^*	*Đ ^a^*	Thermal Transition *T* (°C) *^b^*	Δ*H*_s*i*_ (J/g) *^f^*	*T*_d_ (°C) *^g^*
PEAsA-2	93	8260	1.74	G 18.4 S_x_ 96.4 I *^c^* I 86.1/69.8 S_x_-G *^d,e^*	22.6 *^c^* −12.9, −9.1 *^d^*	260
PEAsA-6	92	13,300	1.23	G 17.3 S_x_ 82.1/99.6 I *^c^* I 61.1 S_x_-G *^d,e^*	34.2, 7.9 *^c^* −27.9 *^d^*	275
PEAsA-12	90	9860	1.98	G 16.8 S_x_ 105.4 I *^c^* I 79.4 S_x_-G *^d,e^*	35.5 *^c^* −34.2 *^d^*	289

*^a^* The number-average molecular weights (*M*_n,GPC_) and molar-mass dispersities (*Đ*) of the acetamides of PEAsA-*n* (i.e., A-PEAsA-*n*, obtained by reacting PEAsA-*n* with acetic anhydride (Scheme S2)) were determined by using a gel permeation chromatograph (GPC) with THF as the eluent (polystyrene standards). *^b^* G = glassy, S_x_ = highly ordered smectic, I = isotropic. *^c^* DSC second heating scan under nitrogen (10 °C min^−1^). *^d^* DSC first cooling scan under nitrogen (−10 °C min^−1^). *^e^* The *T*_g_ data could not be accurately determined because the glass transition steps in the DSC first cooling scan thermograms of the polymers are negligible. *^f^* Enthalpy of the transition between the highly ordered smectic and isotropic phases. *^g^* The temperature at 5% weight loss of the polymers under nitrogen was determined by TGA heating experiments (10 °C min^−1^).

**Table 2:** Mechanical properties and photoinduced stress of the uniaxially oriented PEAsA-*n* fibers.

Sample	Elastic Modulus (MPa)	Rupture Strength (MPa)	Elongation at Break (%)	Photoinduced Stress (kPa)
PEAsA-2 fiber	656.2 ± 26.1	10.9 ± 0.6	1.8 ± 0.1	320.4 ± 17.5
PEAsA-6 fiber	704.4 ± 35.3	20.9 ± 0.9	3.0 ± 0.1	373.2 ± 22.0
PEAsA-12 fiber	749.3 ± 11.4	30.7 ± 0.4	4.3 ± 0.1	295.6 ± 16.3

## Data Availability

Not applicable.

## Supplementary Materials

The following supporting information can be downloaded at https://www.mdpi.com/article/10.3390/molecules28104174/s1, Scheme S1: Chemical structure and synthetic procedure of AAzo-S; Scheme S2: Chemical structures and synthetic procedure of A-PEAsA-*n* (*n* = 2, 6, 12); Figure S1: Schematic of the sample preparation procedure for the XRD measurement of the uniaxially oriented main-chain azo polymer fibers; Figure S2: ^13^C NMR spectra of AAzo-OH (A), M-Azo (B), and PEAsA-6 (C) in CDCl_3_; Figure S3: POM images of PEAsA-*n* (*n* = 2, 6, 12): (a) PEAsA-2 upon cooling to 84 °C (a1) and 68 °C (a2), respectively, and annealing for 30 min (during the first cooling process); (b) PEAsA-6 upon cooling to 59 °C (b1) and annealing for 30 min (during the first cooling process); PEAsA-6 upon heating to 80 °C (b2), 84 °C (b3), 86 °C (b4), 92 °C (b5), and 95 °C (b6), respectively, and annealing for 30 min (during the second heating process); (c) PEAsA-12 upon cooling to 77 °C and annealing for 30 min (during the first cooling process); Figure S4: UV and visible light-induced photoisomerization cycles of the PEAsA-6 thin film at 25 °C; Figure S5: POM images of the textures of a PEAsA-2 fiber (a) and a PEAsA-12 fiber (b) taken at room temperature. Sample angle to the analyzer: *θ* = 0° (left) and *θ* = 45° (right); Figure S6: Polarized UV-vis absorption spectra of the uniaxially oriented PEAsA-2 fiber (a) and PEAsA-12 fiber (b); Figure S7: XRD spectra of the uniaxially oriented PEAsA-*n* (*n* = 2, 12) fibers; Figure S8: (a,c) Photographs of a representative PEAsA-2 fiber (a) or PEAsA-12 fiber (c) that exhibits photoinduced bending and unbending upon exposure to 365 nm of UV light (90 mW cm^−2^) and visible light (λ > 510 nm, 35 mW cm^−2^) at 25 °C (fiber size: 10 mm × 20 μm). (b,d) The reversible deformation of the PEAsA-2 fiber (c) or PEAsA-12 fiber (d) characterized by tracing the bent distance from its straight state at 25 °C; Figure S9: 3D shape reprogramming of a uniaxially oriented PEAsA-2 fiber and photomobile behaviors of the resulting reshaped photoactuator. (a) Reshaping a uniaxially oriented PEAsA-2 fiber (diameter: 24 μm) into a fiber spring (the wire diameter: 5 mm) at 25 °C and its shape (top) and homogeneous azo mesogen alignment (below, the reshaped fiber was untwisted for POM observations) remain unchanged after being kept at 25 or 70 °C for 10 days. (b) Photomobile behaviors of the reshaped fiber spring at room temperature under the irradiation of UV light (365 nm, 40 mW cm^−2^) and visible light (*λ* > 510 nm, 30 mW cm^−2^); Figure S10: 3D shape reprogramming of a uniaxially oriented PEAsA-12 fiber and photomobile behaviors of the resulting reshaped photoactuator. (a) Reshaping a uniaxially oriented PEAsA-12 fiber (diameter: 24 μm) into a fiber spring (the wire diameter: 5 mm) at 25 °C and its shape (top) and homogeneous azo mesogen alignment (below, the reshaped fiber was untwisted for POM observations) remain unchanged after being kept at 25 or 70 °C for 10 days. (b) Photomobile behaviors of the reshaped fiber spring at room temperature under the irradiation of UV light (365 nm, 40 mW cm^−2^) and visible light (*λ* > 510 nm, 30 mW cm^−2^); Figure S11: ^1^H NMR spectra of the original PEAsA-6 (A) and the recycled PEAsA-6 (B); Movie S1: A movie clip that demonstrates the photomobile behaviors of the reshaped PEAsA-6 fiber spring at room temperature under the irradiation of UV light (365 nm, 40 mW cm^−2^) and visible light (*λ* > 510 nm, 30 mW cm^−2^); Movie S2: A movie clip that demonstrates the photomobile behaviors of the reshaped PEAsA-2 fiber spring at room temperature under the irradiation of UV light (365 nm, 40 mW cm^−2^) and visible light (*λ* > 510 nm, 30 mW cm^−2^); Movie S3: A movie clip that demonstrates the photomobile behaviors of the reshaped PEAsA-12 fiber spring at room temperature under the irradiation of UV light (365 nm, 40 mW cm^−2^) and visible light (*λ* > 510 nm, 30 mW cm^−2^).

## References

[1] Ohm C., Brehmer M., Zentel R. (2010). Liquid crystalline elastomers as actuators and sensors. Adv. Mater..

[2] Yu H., Ikeda T. (2011). Photocontrollable liquid-crystalline actuators. Adv. Mater..

[3] Yang H., Ye G., Wang X., Keller P. (2011). Micron-sized liquid crystalline elastomer actuators. Soft Matter.

[4] Broer D.J., Bastiaansen C.M.W., Debije M.G., Schenning A.P.H.J. (2012). Functional organic materials based on polymerized liquid-crystal monomers: Supramolecular hydrogen-bonded systems. Angew. Chem. Int. Ed..

[5] Ube T., Ikeda T. (2014). Photomobile polymer materials with crosslinked liquid-crystalline structures: Molecular design, fabrication, and functions. Angew. Chem. Int. Ed..

[6] White T.J., Broer D.J. (2015). Programmable and adaptive mechanics with liquid crystal polymer networks and elastomers. Nat. Mater..

[7] Bisoyi H.K., Li Q. (2016). Light-driven liquid crystalline materials: From photo-induced phase transitions and property modulations to applications. Chem. Rev..

[8] Jiang Z.C., Xiao Y.Y., Zhao Y. (2019). Shining light on liquid crystal polymer networks: Preparing, reconfiguring, and driving soft actuators. Adv. Opt. Mater..

[9] Pang X., Lv J., Zhu C., Qi L., Yu Y. (2019). Photodeformable azobenzene-containing liquid crystal polymers and soft actuators. Adv. Mater..

[10] Chen M., Liang S., Liu C., Liu Y., Wu S. (2020). Reconfigurable and recyclable photoactuators based on azobenzene-containing polymers. Front. Chem..

[11] Zhang H. (2021). Reprocessable photodeformable azobenzene polymers. Molecules.

[12] Saed M.O., Gablier A., Terentjev E.M. (2022). Exchangeable liquid crystalline elastomers and their applications. Chem. Rev..

[13] Herbert K.M., Fowler H.E., McCracken J.M., Schlafmann K.R., Koch J.A., White T.J. (2022). Synthesis and alignment of liquid crystalline elastomers. Nat. Rev. Mater..

[14] Kondo M., Yu Y., Ikeda T. (2006). How does the initial alignment of mesogens affect the photoinduced bending behavior of liquid-crystalline elastomers?. Angew. Chem. Int. Ed..

[15] Cheng Z., Ma S., Zhang Y., Huang S., Chen Y., Yu H. (2017). Photomechanical motion of liquid-crystalline fibers bending away from a light source. Macromolecules.

[16] Fang L., Zhang H.T., Li Z., Zhang Y., Zhang Y.Y., Zhang H. (2013). Synthesis of reactive azobenzene main-chain liquid crystalline polymers via Michael addition polymerization and photomechanical effects of their supramolecular hydrogen-bonded fibers. Macromolecules.

[17] Nie J., Liu X., Yan Y., Zhang H. (2017). Supramolecular hydrogen-bonded photodriven actuators based on an azobenzene-containing main-chain liquid crystalline poly(ester-amide). J. Mater. Chem. C.

[18] Wang Z., Zhang H. (2020). Synthesis of an azobenzene-containing main-chain crystalline polymer and photodeformation behaviors of its supramolecular hydrogen-bonded fibers. Chin. J. Polym. Sci..

[19] Wang L., Zhou Y., Ma S., Zhang H. (2021). Reprocessable and healable room temperature photoactuators based on a main-chain azobenzene liquid crystalline poly(ester-urea). J. Mater. Chem. C.

[20] Zhou Y., Wang L., Ma S., Zhang H. (2022). Fully room-temperature reprogrammable, reprocessable, and photomobile soft actuators from a high-molecular-weight main-chain azobenzene crystalline poly(ester-amide). ACS Appl. Mater. Interfaces.

[21] Zhou Y., Wang L., Zhang H. (2022). Enhancing the performances of physically cross-linked photodeformable main-chain azobenzene poly(ester-amide)s via chemical structure engineering. Polym. Chem..

[22] Ube T., Nakayama R., Ikeda T. (2022). Photoinduced motions of thermoplastic polyurethanes containing azobenzene moieties in main chains. Macromolecules.

[23] Zhang P., Lan Z., Wei J., Yu Y. (2021). Photodeformable azobenzene-containing polyimide with flexible linkers and molecular alignment. ACS Macro Lett..

[24] Zhong H.-Y., Chen L., Yang R., Meng Z.-Y., Ding X.-M., Liu X.-F., Wang Y.-Z. (2017). Azobenzene-containing liquid crystalline polyester with π-π interactions: Diverse thermo- and photo-responsive behaviours. J. Mater. Chem. C.

[25] de Gennes P.-G., Hébert M., Kant R. (1997). Artificial muscles based on nematic gels. Macromol. Symp..

[26] Aly K.I., Abdel-Rahman M.A., Hussein M.A. (2010). New polymer syntheses Part 53. Novel polyamides of diarylidenecycloalkanone containing azo groups in the polymer backbone: Synthesis and characterization. Int. J. Polym. Mater.

[27] Mather B.D., Viswanathan K., Miller K.M., Long T.E. (2006). Michael addition reactions in macromolecular design for emerging technologies. Prog. Polym. Sci..

[28] Cheng W., Wu D., Liu Y. (2016). Michael addition polymerization of trifunctional amine and acrylic monomer: A versatile platform for development of biomaterials. Biomacromolecules.

[29] Ube T., Kawasaki K., Ikeda T. (2016). Photomobile liquid-crystalline elastomers with rearrangeable networks. Adv. Mater..

[30] Jiang Z.C., Xiao Y.Y., Yin L., Han L., Zhao Y. (2020). “Self-lockable” liquid crystalline Diels-Alder Dynamic network actuators with room temperature programmability and solution reprocessability. Angew. Chem. Int. Ed..

[31] Huang S., Shen Y., Bisoyi H.K., Tao Y., Liu Z., Wang M., Yang H., Li Q. (2021). Covalent adaptable liquid crystal networks enabled by reversible ring-opening cascades of cyclic disulfides. J. Am. Chem. Soc..

[32] Yoon Y., Ho R.-M., Li F., Leland M.E., Park J.-Y., Cheng S.Z.D., Percec V., Chu P. (1997). Existence of highly ordered smectic structures in a series of main-chain liquid-crystalline polyethers. Prog. Polym. Sci..

[33] Jeong K.U., Knapp B.S., Ge J.J., Jin S., Graham M.J., Xiong H.M., Harris F.W., Cheng S.Z.D. (2005). Structures and phase transformations of asymmetric bent main-chain liquid crystalline polyesters. Macromolecules.

[34] Yu Z.-Q., Li T.-T., Zhang Z., Liu J.-H., Yuan W.Z., Lam J.W.Y., Yang S., Chen E.-Q., Tang B.Z. (2015). Phase behaviors of side-chain liquid crystalline polyacetylenes with different length of spacer: Where will the decoupling effect appear?. Macromolecules.

[35] Niemann M., Ritter H. (1993). Comb-like methacrylamide polymers containing condensates of amino acids and azobenzene moieties in the side chains. Makromol. Chem..

[36] Li X., Wen R., Zhang Y., Zhu L., Zhang B., Zhang H. (2009). Photoresponsive side-chain liquid crystalline polymers with an easily cross-linkable azobenzene mesogen. J. Mater. Chem..

[37] Li X., Fang L., Hou L., Zhu L., Zhang Y., Zhang B., Zhang H. (2012). Photoresponsive side-chain liquid crystalline polymers with amide group-substituted azobenzene mesogens: Effects of hydrogen bonding, flexible spacers, and terminal tails. Soft Matter.

[38] Wang G., Tong X., Zhao Y. (2004). Preparation of azobenzene-containing amphiphilic diblock copolymers for light-responsive micellar aggregates. Macromolecules.

[39] Akiyama H., Tamaoki N. (2007). Synthesis and photoinduced phase transitions of poly(*N*-isopropylacrylamide) derivative functionalized with terminal azobenzene units. Macromolecules.

[40] Li M.H., Keller P., Li B., Wang X.G., Brunet M. (2003). Light-driven side-on nematic elastomer actuators. Adv. Mater..

[41] Naciri J., Srinivasan A., Jeon H., Nikolov N., Keller P., Ratna B.R. (2003). Nematic elastomer fiber actuator. Macromolecules.

[42] Yoshino T., Kondo M., Mamiya J.-I., Kinoshita M., Yu Y., Ikeda T. (2010). Three-dimensional photomobility of crosslinked azobenzene liquid-crystalline polymer fibers. Adv. Mater..

[43] Petr M., Katzman B.-A., DiNatale W., Hammond P.T. (2013). Synthesis of a new, low-*T*_g_ siloxane thermoplastic elastomer with a functionalizable backbone and its use as a rapid, room temperature photoactuator. Macromolecules.

[44] Li S., Tu Y., Bai H., Hibi Y., Wiesner L.W., Pan W., Wang K., Giannelis E.P., Shepherd R.F. (2019). Simple synthesis of elastomeric photomechanical switches that self-heal. Macromol. Rapid Commun..

[45] Chen M., Yao B., Kappl M., Liu S., Yuan J., Berger R., Zhang F., Butt H.-J., Liu Y., Wu S. (2020). Entangled azobenzene-containing polymers with photoinduced reversible solid-to-liquid transitions for healable and reprocessable photoactuators. Adv. Funct. Mater..

[46] Peng K., Nain A., Mirzaeifar R. (2019). Tracking the origins of size dependency in the mechanical properties of polymeric nanofibers at the atomistic scale. Polymer.

[47] Hu W. (2013). Principles of Polymer Crystallization.

[48] Yu Y., Maeda T., Mamiya J.-I., Ikeda T. (2007). Photomechanical effects of ferroelectric liquid-crystalline elastomers containing azobenzene chromophores. Angew. Chem. Int. Ed..

