# Supplementary material for: Fully Room Temperature Reprogrammable, Recyclable, and Photomobile Soft Actuators from Physically Cross-Linked Main-Chain Azobenzene Liquid Crystalline Polymers
Source: Molecules. 2023 May 18;28(10):4174. doi: 10.3390/molecules28104174 (PMC10221483; doi:10.3390/molecules28104174)
Supplement: Supplementary file 1 [file molecules-28-04174-s001.zip › molecules-2393755-supplementary.pdf]

Supporting Information

Fully Room Temperature Reprogrammable, Recyclable, and Photomobile Soft Actuators from Physically Cross-Linked Main-Chain Azobenzene Liquid Crystalline Polymers

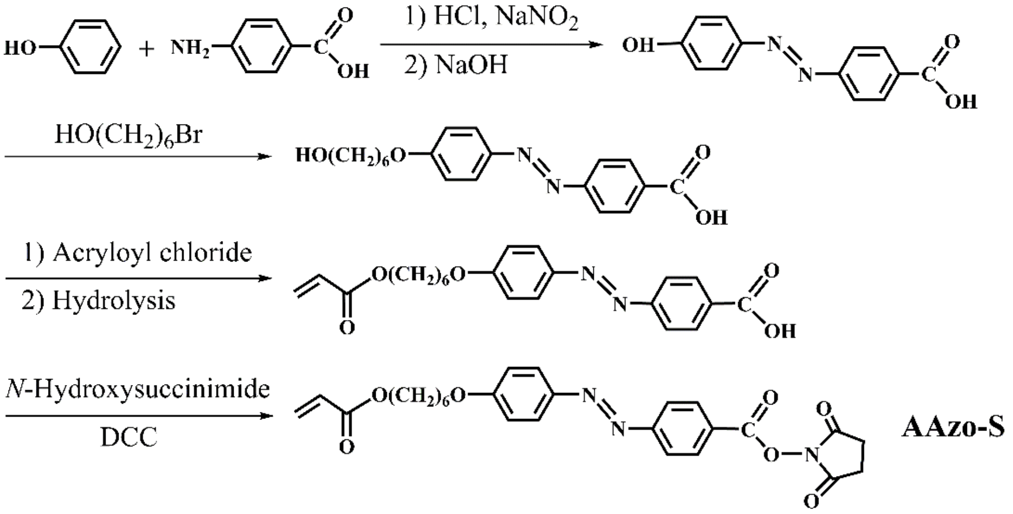

Scheme S1. Chemical structure and synthetic procedure of AAzo-S.

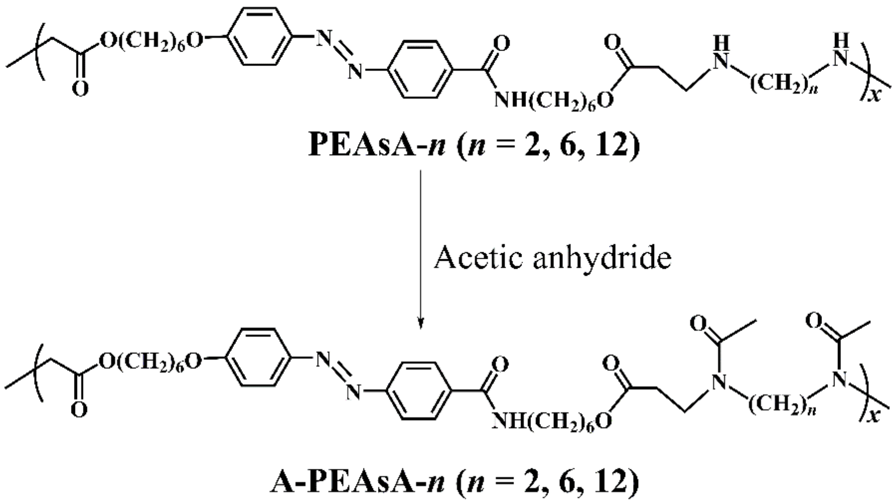

Scheme S2. Chemical structures and synthetic procedure of A-PEAsA-*n* (*n* =2, 6, 12).

Preparation of the thin PEAsA-6 film for the photoresponsivity study

A thin PEAsA-6 film was cast from a PEAsA-6 solution in hexafluoroisopropanol (4.0 mg mL^{-1}, 100 μL) on a clean quartz glass plate (12 × 45 mm). After the solvent was evaporated slowly at ambient temperature for 12 h, a transparent light yellow film was formed on the quartz glass

plate. The thickness (*l*) of the polymer film is estimated to be 740 nm by using the equation $l = VC/\rho S$, where V is the volume of the polymer solution cast on the quartz glass plate, C is the concentration of the polymer solution, ρ is the density of the soild polymer film (ρ is assumed to be 1 g mL^{-1}), and S is the surface area of the quartz glass plate. The photoresponsivity of the resulting thin PEAsA-6 film was studied by first irradiating it with 365 nm UV light (90 mW cm^{-2}) until its photostationary state was reached. The photostationary PEAsA-6 film was then irradiated with visible light (λ > 510 nm, 35 mW cm^{-2}). The UV-vis spectra of the sample were recorded during the above studies. The used UV light and visible light were obtained from a high-pressure mercury lamp (USHIO SP-7, glass filters were used to obtain the desired light).

The detailed sample preparation methods for the XRD characterization of both the azo polymer powders and the uniaxially oriented azo polymer fibers

The sample preparation procedure for measuring the XRD spectra of the polymer powders involved first heating the polymers to the temperatures above their anisotropic temperatures (+10 °C min^{-1}), then cooling them to the temperatures that are 2 °C below the exothermic peak temperatures in their DSC thermograms during the first cooling process (-10 °C min^{-1}) [for PEAsA-6, another two samples were also prepared for XRD measurements, and their preparation involved first heating PEAsA-6 to a temperature above its anisotropic temperature (+10 °C min^{-1}), then cooling it to -50 °C (-10 °C min^{-1}), further heating the sample again to a temperature of 80 and 95 °C, respectively], annealing them at those temperatures for 30 min, and then putting them into liquid nitrogen to preserve their phase structures (all the above heating and cooling were performed under nitrogen atmosphere).

The sample preparation procedure for measuring the XRD spectra of the uniaxially oriented PEAsA-*n* fibers is schematically illustrated in Figure S1. A home-made sample holder was first prepared by cutting an empty rectangle (width = 0.5 cm, length = 1.0 cm) on a thin square-shaped cardboard (width and length = 1.6 cm) (Figure S1A). The azo polymer fibers were then wound around the empty rectangle (parallel to the long axis of the rectangle, as shown in Figure S1B) until the density of the fibers was so high that the objects behind the fibers became invisible (Figure S1C). The sample holder with the wound fibers was then put inside the sample cell for XRD measurement.

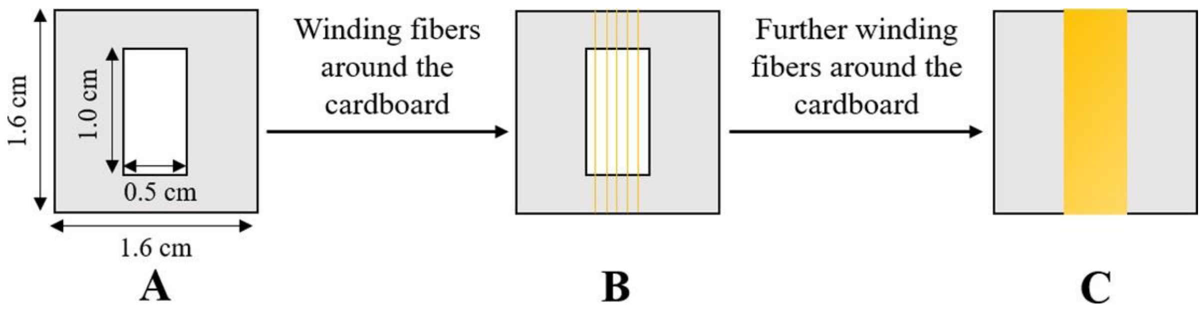

Figure S1. Schematic of the sample preparation procedure for the XRD measurement of the uniaxially oriented main-chain azo polymer fibers.

Determination of the molecular weights and molar-mass dispersities of the polymers

The molecular weights and molar-mass dispersities (*Đ*) of the polymers were determined with a GPC equipped with a Waters 717 plus autosampler, a Waters 515 HPLC pump, three Waters UltraStyragel columns with 5k-600k, 500-30k, and 100-10k molecular ranges, and an online Waters 2414 refractive index detector maintained at 35 °C. THF was used as the eluent at a flow rate of 1.0 mL min^{-1}. The calibration curve was obtained by using polystyrene standards.

Determination of the order parameters of the uniaxially oriented PEAsA-n fibers

The order parameters (*S*) of the uniaxially oriented PEAsA-*n* fibers were determined through measuring their polarized absorption spectra by using a UV-vis scanning spectrophotometer (TU1900, Beijing Purkinje General Instrument Co., Ltd) equipped with a Glan-Taylor prism attachment. The *S* values of the uniaxially oriented fibers were derived from the equation: $S = (A_{//}-A_{\perp})/(A_{//}+2A_{\perp})$, where $A_{//}$ and $A_{\perp}$ represent the absorbance (at 500 nm) obtained with incident light polarized parallel and perpendicular to the uniaxially oriented fibers, respectively.

The sample preparation procedure for measuring the order parameters of the uniaxially oriented PEAsA-*n* fibers is similar as that for the XRD measurement of the fibers except that the sizes of the cardboard and the empty rectangle are different from those shown in Figure S1. A home-made sample holder for polymer fibers was first prepared by cutting an empty rectangle (width = 0.5 cm, length = 3.0 cm) on a thin square-shaped cardboard (width = 1.0 cm, length = 4.0 cm). The azo polymer fibers were then wound around the empty rectangle (parallel to the long axis of the rectangle) until the density of the fibers was so high that the objects behind the fibers became invisible. The sample holder with the wound fibers was then put inside the sample cell for measuring the polarized UV absorption spectra.

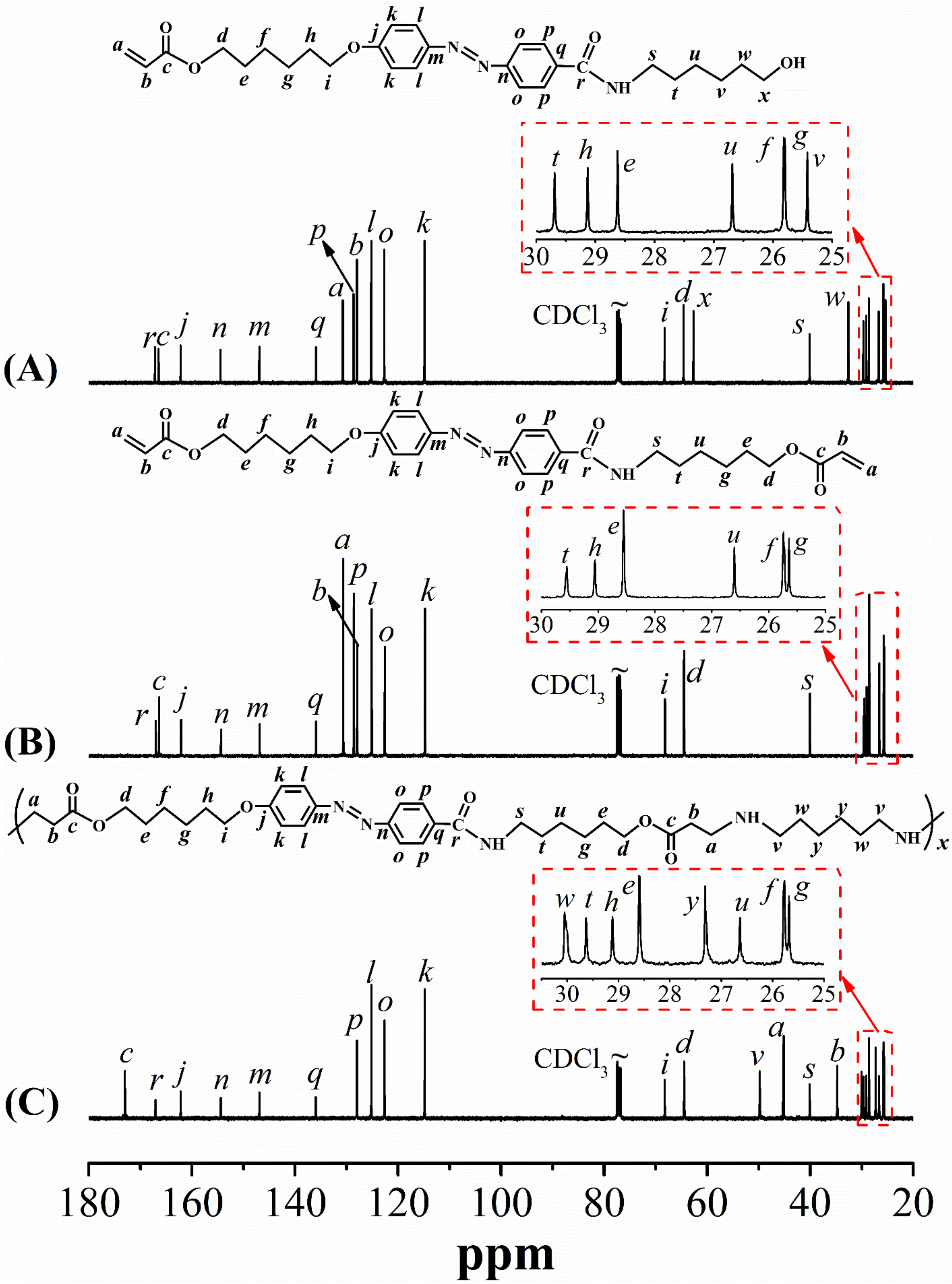

Figure S2. ^{13}C NMR spectra of AAzo-OH (A), M-Azo (B), and PEAsA-6 (C) in $CDCl_3$.

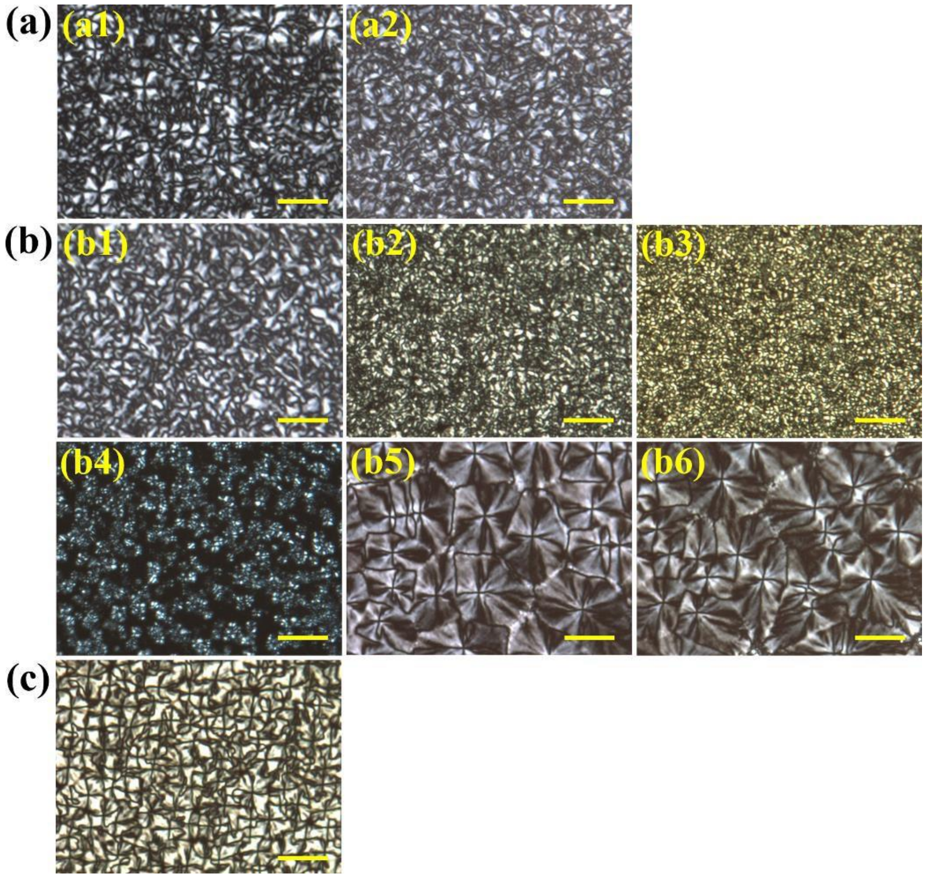

Figure S3. POM images of PEAsA-*n* (*n* =2, 6, 12): (a) PEAsA-2 upon cooling to 84 ^{o}C (a1) and 68 ^{o}C (a2), respectively, and annealing for 30 min (during the first cooling process); (b) PEAsA-6 upon cooling to 59 ^{o}C (b1) and annealing for 30 min (during the first cooling process); PEAsA-6 upon heating to 80 ^{o}C (b2), 84 ^{o}C (b3), 86 ^{o}C (b4), 92 ^{o}C (b5), and 95 ^{o}C (b6), respectively, and annealing for 30 min (during the second heating process); (c) PEAsA-12 upon cooling to 77 ^{o}C and then annealed for 30 min (during the first cooling process). The scale bar is 20 μm.

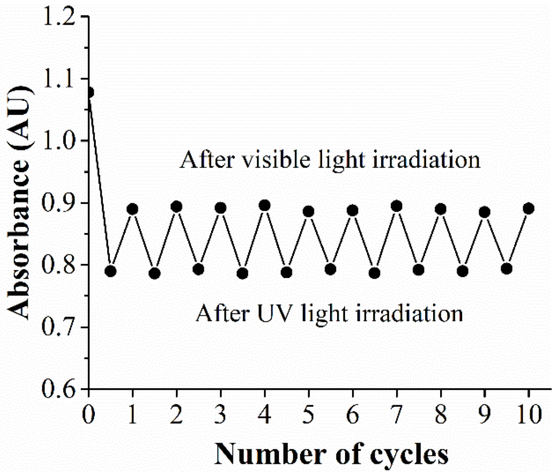

Figure S4. UV and visible light-induced photoisomerization cycles of the PEAsA-6 thin film at 25 °C.

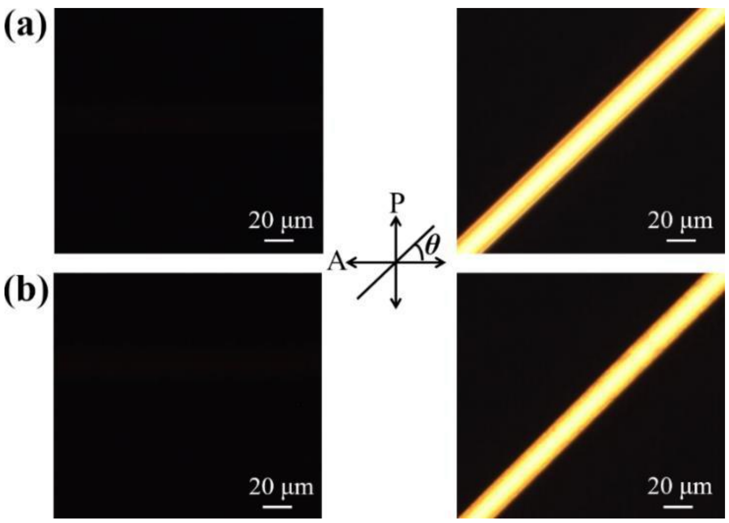

Figure S5. POM images of the textures of a PEAsA-2 fiber (a) and a PEAsA-12 fiber (b) taken at room temperature. Sample angle to the analyzer: θ = 0º (left) and θ = 45º (right).

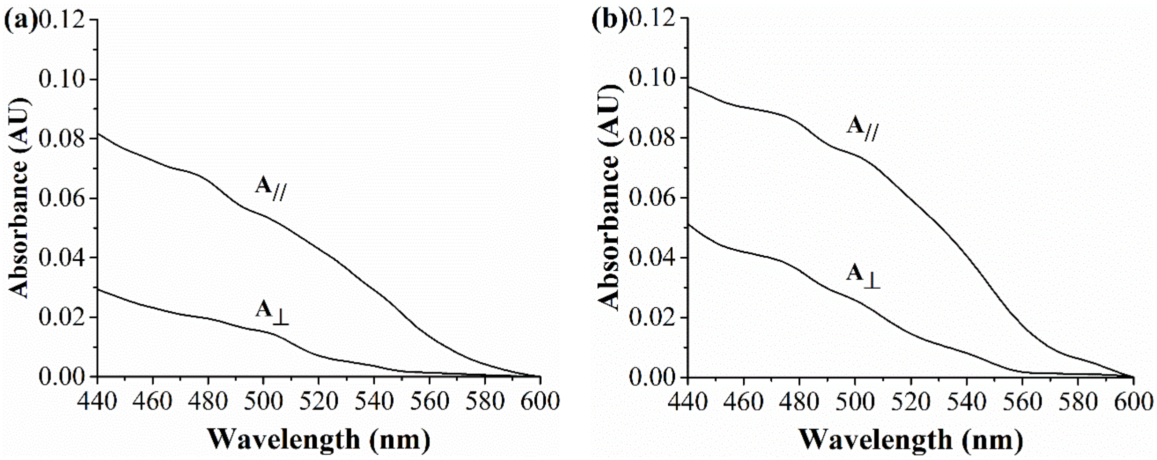

Figure S6. Polarized UV-vis absorption spectra of the uniaxially oriented PEAsA-2 fiber (a) and PEAsA-12 fiber (b).

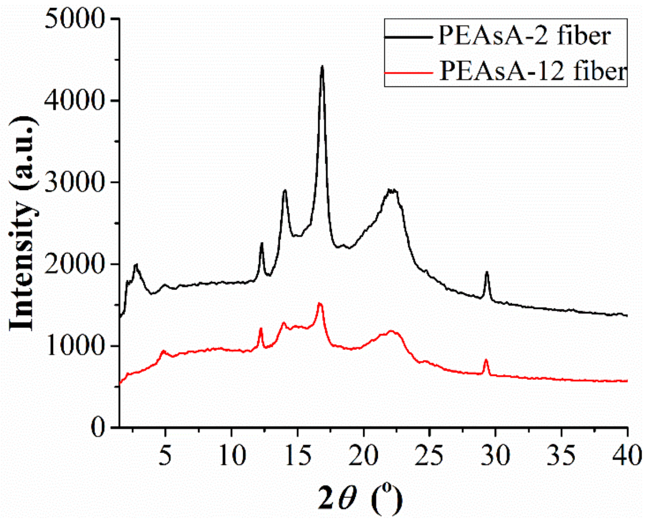

Figure S7. XRD spectra of the uniaxially oriented PEAsA-*n* (*n* = 2, 12) fibers.

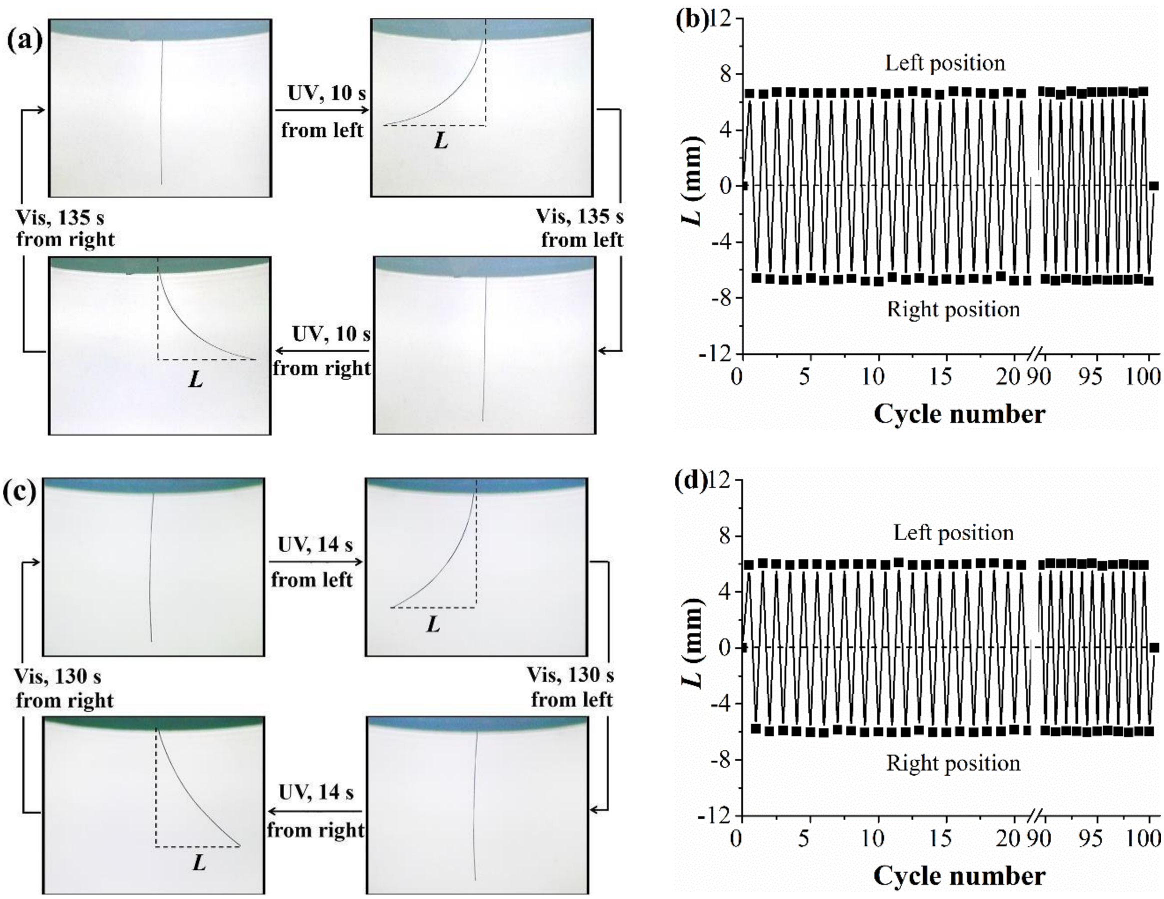

Figure S8. (a,c) Photographs of a representative PEAsA-2 fiber (a) or PEAsA-12 fiber (c) that exhibits photoinduced bending and unbending upon exposure to 365 nm of UV light (90 mW cm^{-2}) and visible light (λ > 510 nm, 35 mW cm^{-2}) at 25 ^{o}C (fiber size: 10 mm × 20 μm. (b,d) The reversible deformation of the PEAsA-2 fiber (c) or PEAsA-12 fiber (d) characterized by tracing the bent distance from its straight state at 25 °C.

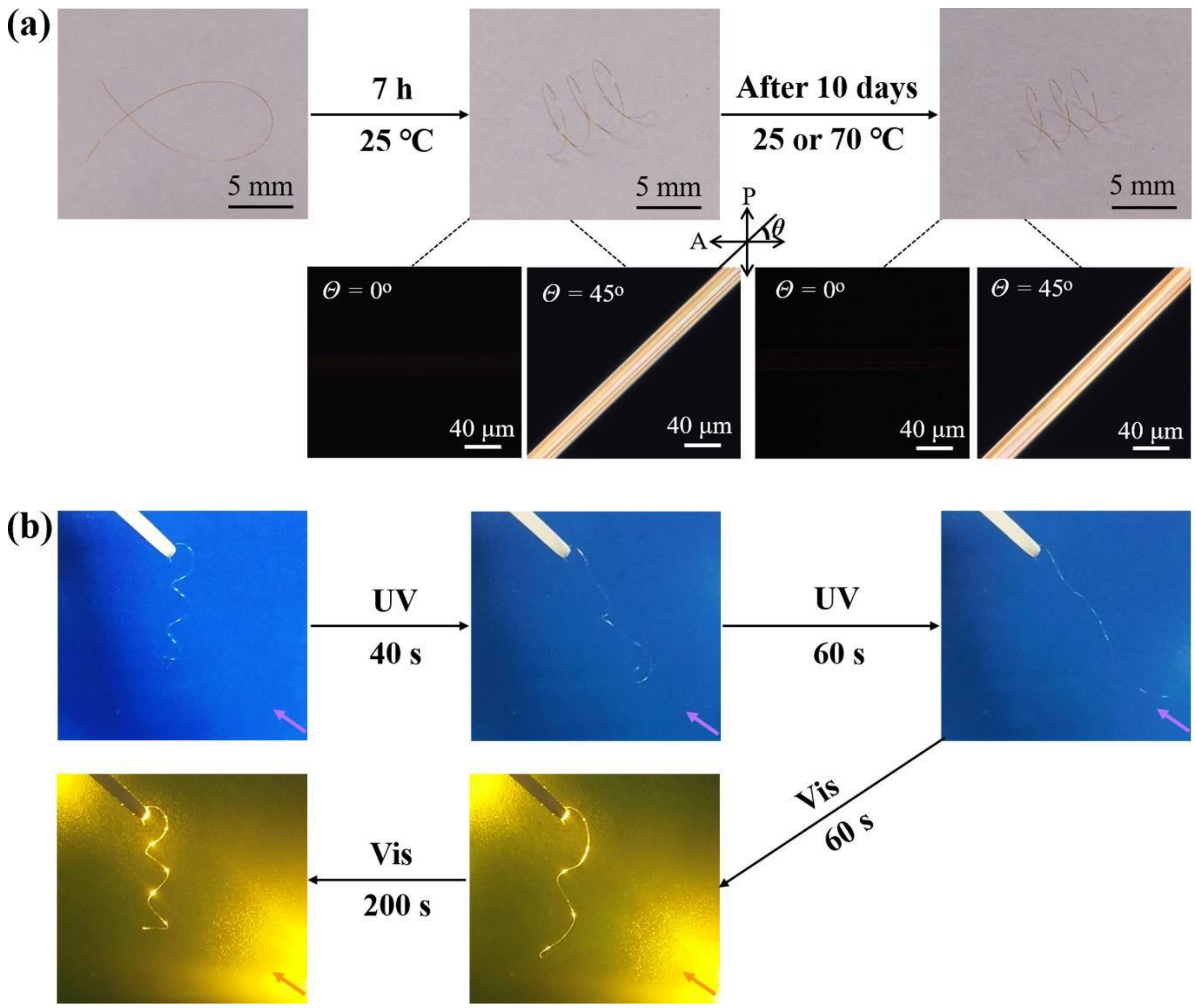

Figure S9. 3D shape reprogramming of a uniaxially oriented PEAsA-2 fiber and photomobile behaviors of the resulting reshaped photoactuator. (a) Reshaping a uniaxially oriented PEAsA-2 fiber (diameter: 24 μm) into a fiber spring (the wire diameter: 5 mm) at 25 °C and its shape (top) and homogeneous azo mesogen alignment (below, the reshaped fiber was untwisted for POM observations) remain unchanged after being kept at 25 or 70 °C for 10 days. (b) Photomobile behaviors of the reshaped fiber spring at room temperature under the irradiation of UV light (365 nm, 40 mW cm^{-2}) and visible light (λ > 510 nm, 30 mW cm^{-2}).

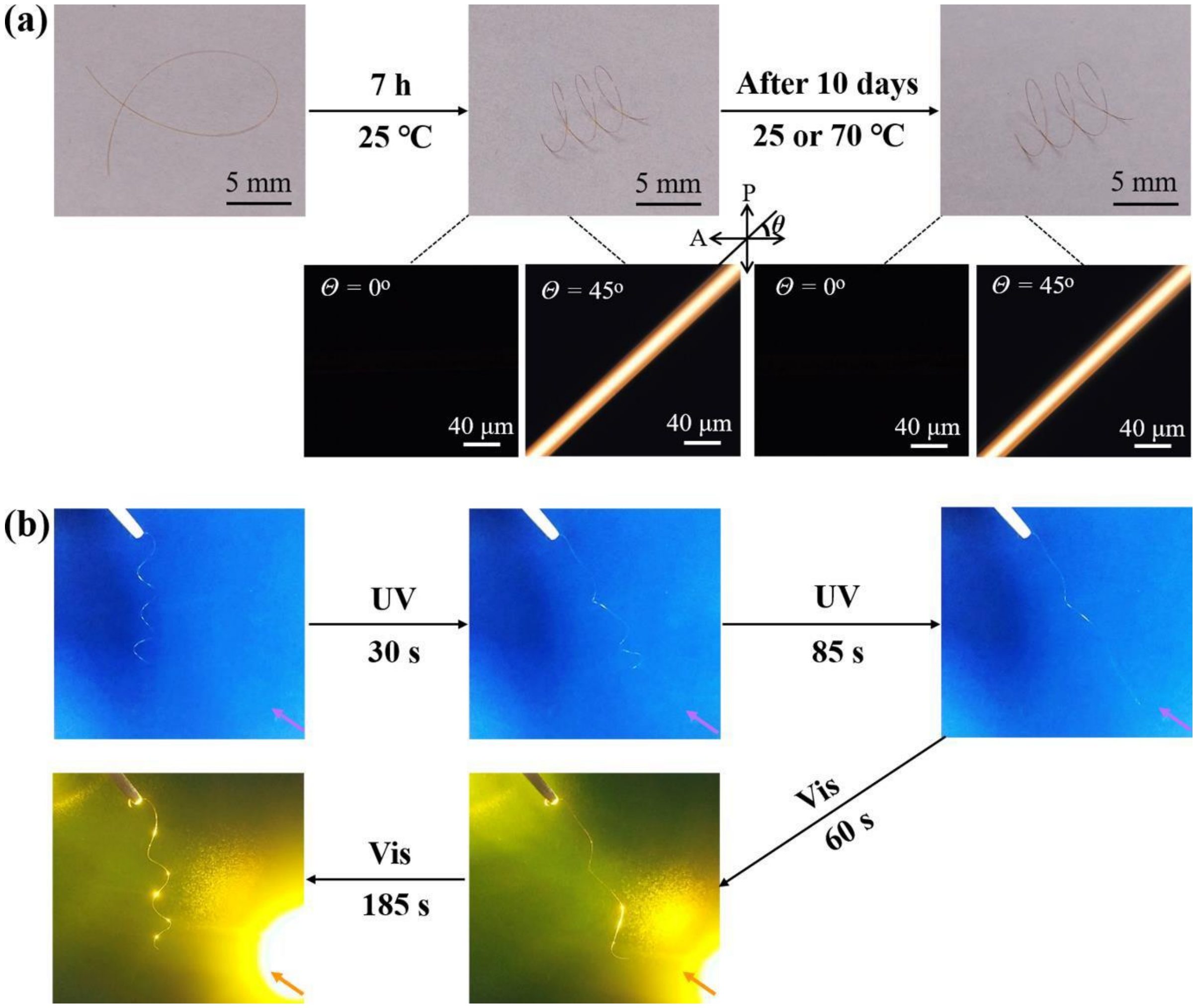

Figure S10. 3D shape reprogramming of a uniaxially oriented PEAsA-12 fiber and photomobile behaviors of the resulting reshaped photoactuator. (a) Reshaping a uniaxially oriented PEAsA-12 fiber (diameter: 24 μm) into a fiber spring (the wire diameter: 5 mm) at 25 ^{o}C and its shape (top) and homogeneous azo mesogen alignment (below, the reshaped fiber was untwisted for POM observations) remain unchanged after being kept at 25 or 70 ^{o}C for 10 days. (b) Photomobile behaviors of the reshaped fiber spring at room temperature under the irradiation of UV light (365 nm, 40 mW cm^{-2}) and visible light (λ > 510 nm, 30 mW cm^{-2}).

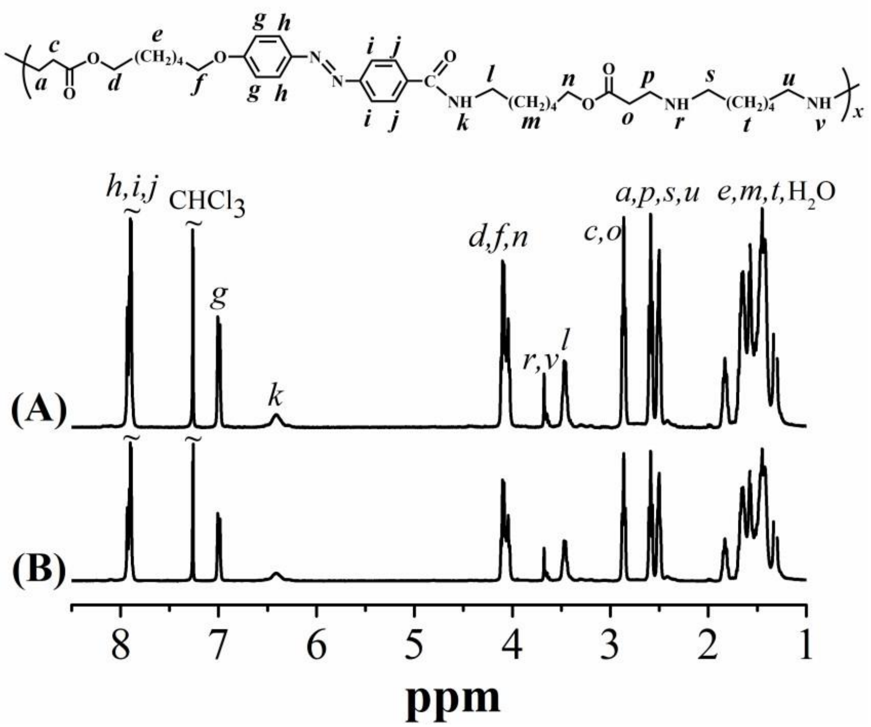

Figure S11. ^{1}H NMR spectra of the original PEAsA-6 (A) and the recycled PEAsA-6 (B).

Supplementary Videos

Movie S1. A movie clip that demonstrates the photomobile behaviors of the reshaped PEAsA-6 fiber spring at room temperature under the irradiation of UV light (365 nm, 40 mW cm^{-2}) and visible light (λ > 510 nm, 30 mW cm^{-2}).

Movie S1.mp4

Movie S2. A movie clip that demonstrates the photomobile behaviors of the reshaped PEAsA-2 fiber spring at room temperature under the irradiation of UV light (365 nm, 40 mW cm^{-2}) and visible light (λ > 510 nm, 30 mW cm^{-2}).

Movie S2.mp4

Movie S3. A movie clip that demonstrates the photomobile behaviors of the reshaped PEAsA-12 fiber spring at room temperature under the irradiation of UV light (365 nm, 40 mW cm^{-2}) and visible light (λ > 510 nm, 30 mW cm^{-2}).

Movie S3.mp4
